# Imogene: identification of motifs and *cis*-regulatory modules underlying gene co-regulation

**DOI:** 10.1093/nar/gku209

**Published:** 2014-03-25

**Authors:** Hervé Rouault, Marc Santolini, François Schweisguth, Vincent Hakim

**Affiliations:** 1 Developmental and Stem Cell Biology Department, Institut Pasteur, F-75015 Paris, France; 2 CNRS, URA2578, F-75015 Paris, France; 3 Laboratoire de Physique Statistique, CNRS, École Normale Supérieure, Université P. et M. Curie, Université Paris-Diderot

## Abstract

*Cis*-regulatory modules (CRMs) and motifs play a central role in tissue and condition-specific gene expression. Here we present *Imogene*, an ensemble of statistical tools that we have developed to facilitate their identification and implemented in a publicly available software. Starting from a small training set of mammalian or fly CRMs that drive similar gene expression profiles, *Imogene* determines *de novo* *cis*-regulatory motifs that underlie this co-expression. It can then predict on a genome-wide scale other CRMs with a regulatory potential similar to the training set. *Imogene* bypasses the need of large datasets for statistical analyses by making central use of the information provided by the sequenced genomes of multiple species, based on the developed statistical tools and explicit models for transcription factor binding site evolution. We test *Imogene* on characterized tissue-specific mouse developmental CRMs. Its ability to identify CRMs with the same specificity based on its *de novo* created motifs is comparable to that of previously evaluated ‘motif-blind’ methods. We further show, both in flies and in mammals, that *Imogene de novo* generated motifs are sufficient to discriminate CRMs related to different developmental programs. Notably, purely relying on sequence data, *Imogene* performs as well in this discrimination task as a previously reported learning algorithm based on Chromatin Immunoprecipitation (ChIP) data for multiple transcription factors at multiple developmental stages.

## INTRODUCTION

The identification and functional characterization of the non-coding sequences that direct the spatio-temporal specificity of gene expression in eukaryotes is of fundamental importance in developmental biology (1) and can find crucial applications in medicine (2).These regulatory sequences are generally located distally from gene promoters and termed enhancers or more generically *cis*-regulatory modules (CRMs) since they can either enhance or repress gene expression (3). They usually are of the order of 500 nucleotides (nts) long and can be located as far as several mega base-pairs away from the transcription start sites (TSSs) of the genes that they regulate. CRMs are composed of transcription factor binding sites (TFBSs) that bring spatio-temporal specificity to the expression of their target promoters (4). Detailed studies in both flies and vertebrates (5) have shown that CRMs contain multiple binding sites for transcription factors (TFs) that can be either identical (homotypic clustering) or different (heterotypic clustering). Homotypic clustering can provide cooperative TF binding and sharp on-off gene expression whereas heterotypic clustering allows for combinatorial gene regulation. The extent to which the order and relative positioning of the different TFBSs in CRMs matter remains however debated (6,7).

With the advent of ChIP-seq techniques, genome-wide studies are providing large amount of data on the binding loci of tissue-specific TFs (8), as well as on other factors that regulate transcription, e.g. by modifying chromatin structure (p300, CTCF, histone marks, etc.) (9,10). These protein binding data have helped the identification of numerous CRMs specific to well-defined developmental processes and it has brought important information on CRM structure. However, genome-wide studies suffer from limitations. A full characterization of regulatory mechanisms would require ChIP-seq analysis to be performed for every potential regulatory factor, on every tissue, at multiple developmental stages. The results would also have to be obtained for the often heterogeneous cells that constitute the tissue of interest instead of being averaged over them as it usually needs to be the case. Finally, and very importantly, binding cannot be equated to functional regulation.

Therefore, *in silico* identification of CRMs forms a useful complement to genome-wide binding studies. Classic case-by-case studies or large-scale binding data (11), as previously described, often provide a moderate number (about ten to a few tens) of CRMs, active in the co-regulation of a subset of genes, in specific biological systems or in the formation of different organs at various stages of development. Identifying the important binding sites on these known sequences would help to bypass some of the limitations of large-scale studies by providing information on the factor involved, both known and new, as well as on the existence of a regulatory grammar (12). It should also help one to determine other CRMs providing specific expression patterns, a difficult task at present given the absence of close association (13) between CRMs and their target genes in higher eukaryotes. These labor-intensive experimental tasks could be eased by computational work. To this end, we have previously developed (14) statistical tools to determine *cis*-regulatory elements *de novo* in a set of input DNA sequences encoding a common transcriptional regulation. They allow the determination of regulatory elements from input DNA sequences without any prior information on the TFs acting in *cis* or on their binding sites. They make central use of the phylogenetic information contained in the aligned DNA sequences of related species. The method was applied to the *Drosophila melanogaster* gene expression program in sensory organ precursor cells (SOPs), a specific type of neural progenitor cells (14). Predicted motifs included already characterized TFBS as well as new motifs and were successfully tested by mutational analysis. These motifs were used to rank intergenic DNA fragments genome-wide for their regulatory potential in SOPs. Of the top 29 predicted CRMs, 38% were found by transgenic assays to direct transcription in SOP. A larger fraction (65%) drove more generally transcription in neural precursors.

This successful application to a *Drosophila* transcriptional program led us to try and extend the method developed in (14) to the case of mammalian CRMs. The task of determining *cis*-regulatory elements is even more difficult for mammalian genomes than for *Drosophila* ones since they are an order of magnitude richer in intergenic sequences (15,16). To tackle this challenge, we have developed *Imogene*, a computer algorithm and software that we present here and characterize. *Imogene* predicts:

1. *cis*-regulatory sequences (of about 10 nt long) within a moderate set size of 10–30 CRMs, responsible for specific gene co-regulation, as well as a set of probability weight matrices (PWMs) or motifs (17,18) characterizing the DNA-binding specificity of the associated putative factors.
2. novel CRMs at the genomic scale with the same expression pattern as the starting set of CRMs, based on the set of built PWMs.

Numerous algorithms have already been developed to try and map *cis*-underlying transcriptional regulation (see, e.g. (3,17,19–21) for recent reviews). *Imogene* differs from previous methods in several respects. *Imogene* aim is most similar to the goal of the ‘motif-blind’ algorithms analyzed in (22). These algorithms have been specially designed to characterize the specificity of a small set of CRMs, contrary to other algorithms that are aimed at the analysis of large datasets such as whole ChIP-seq peak regions (23). As *Imogene*, they work *de novo* instead of using already characterized binding motifs (24–32). Faced to the weak statistical discriminative power offered by the starting set of characterized CRMs, the algorithms of (22) try and distinguish regulatory sequences by their entire content in short nucleotide sequences as also proposed in other works (33–37). On the contrary, *Imogene* insists on building *cis*-regulatory motifs since those are important for experimental work. It instead relies on conservation and the comparison of multiple sequenced genomes.

In the following, the general methodology of *Imogene* is first presented. Then, *Imogene* performance on mammalian CRMs is assessed. Imogene is trained on CRMs pertaining to neural tube and limb developmental programs during embryogenesis. It is shown to successfully classify other CRMs in the same class based on its *de novo* created list of best motifs that contained both new and already known motifs. *Imogene* is furthermore found to perform comparably to ‘motif-blind’ algorithms using the benchmark and methodology of (22). We then consider the distinct but related task of discriminating CRMs with different specificities, rather than discriminating a set of specific CRMs from background intergenic sequences. *Imogene* is shown to accurately discriminate mammalian neural tube from limb CRMs on the basis of very few learned motifs. To further assess the performance of *Imogene*, it is applied to the discrimination of five sets of mesodermal fly CRMs, a task previously considered in (38). Remarkably, the CRM classification solely based on *Imogene* *de novo* generated motifs is found to be of similar quality as the results obtained in (38) based on ChIP binding data for multiple TFs at several developmental time points. Finally, the developed publicly available *Imogene* interface is presented.

## MATERIALS AND METHODS

### Genome alignments

The alignments were downloaded from ftp://ftp.ensembl.org/pub/release-63/emf/ensembl-compara/epo_12_eutherian for mammals and from http://www.biostat.wisc.edu/ cdewey/fly_CAF1/data for *Drosophilae*. For the latter case, we have used the alignments engineered by A. Caspi with the help of the Mercator and MAVID programs. In both cases, the alignments were processed through a customized script to produce alignments in FASTA format, mask for coding sequences (CDS) and simple repeats (see below). These scripts are available in the *Imogene* distribution.

### Annotations

The CDS coordinates were downloaded from ftp://ftp.ensembl.org/pub/release-64/gtf/mus_musculus for mammals (mm9 coordinates) and from ftp://ftp.flybase.net/releases/FB2011_06/dmel_r5.38/gff for *Drosophilae* (release 5 coordinates). In the case of mammals, the TSS coordinates were obtained separately from http://hgdownload.cse.ucsc.edu/goldenPath/mm9/database. Mammalian alignments were already masked for repeat sequences. *Drosophilae* alignments were masked using the coordinates indicated in the *gff* file.

### Phylogenetic trees

The phylogenetic trees used within *Imogene* are displayed in Supplementary Figure S1. For Drosophilae, the distances are taken from Heger and Ponting (39). For mammals, they are obtained from the Ensembl (15) website (www.ensembl.org).

### Background sequences

*Imogene* computes the statistical over-representation of the predicted motifs by comparing them to 20 Mb of background intergenic DNA (10^4^ regions of 2 kb). The script that generates the random coordinates is included in the distribution of *Imogene* as well as the actual coordinates of the produced intergenic regions.

### Training sets

The two used mammalian training sets (limb and neural tube) were obtained from http://enhancer.lbl.gov based on the work of Visel *et al.* (11). They were manually curated to produce a high-quality dataset, with respectively 41 CRMs for the limb and 33 for the neural tube. We further pruned out uninformative CRMs for which no motifs could be generated, either because of repeat masking or because of lack of conservation. More precisely, the reference species sequence was scanned using a window size corresponding to the motif size. If a sequence did not contain any masked nucleotide, we looked in the other species for any unmasked sequence in the surrounding neighborhood of ±20 nt, our flexibility criterion when defining a conserved instance. If putative orthologous sequences were found in enough species to satisfy our conservation requirements (see below), the site was declared as a putative conserved site for a regulatory motif. This filtering step resulted in final sets of 39 limb CRMs (minimal length 789 bp, maximal length 9052 bp and average length 3045 bp) and 29 neural tube CRMs (minimal length 585 bp, maximal length 3045 bp and average length 2419 bp).

The *Drosophilae* training sets were obtained from (38). Coordinate files are given as Supplementary Material.

### Main program

The main program is written in C++ and adapted from the program used in a previous study (14). It is distributed under the GNU GPL license and available as a git repository at http://github.com/hrouault/Imogene. The user manual is available at http://hrouault.github.io/Imogene/. The program can be accessed through a web interface at http://mobyle.pasteur.fr/cgi-bin/portal.py#forms::imogene.

### Binding site scores

A given motif is represented by a PWM with frequency $w$_*i*, *b*_ for the base *b* at position *i*. The index *i* runs from 1 to *l*_*m*_, the size of the motif, which is a parameter in the program that takes the same value for all considered motifs. The binding score of a sequence *s*_*i*_ for such a motif is defined through the corresponding PWM as:

(1) \begin{equation*} S=\sum _{i}\log _2\left(\frac{ w_{i,s_i}}{\pi _{s_i}}\right), \end{equation*}

where π_*b*_ is the mean frequency of the base *b* within intergenic regions ($\pi _\textrm {A,T}=0.30$ and $\pi _\textrm {C,G}=0.20$) as measured on the ‘background sequences’ (see the ‘Background sequences’ subsection for their detailed description). A sequence is considered as a binding site in the reference species (*D. melanogaster* or *Mus musculus*) when its score *S* is larger than the score threshold (*S*_*s*_ or *S*_*g*_) defined by the user of *Imogene*.

### Conservation requirements for binding sites

*Imogene* iteratively builds PWM from binding sites that have conserved instances in different species. The conservation requirement is that orthologous instances are found in at least three distant species, including the reference species. For mammals, the six groups of related species are composed of: *M. musculus* and *Rattus norvegicus*; *Callithrix jacchus*, *Macaca mulatta*, *Pongo abelii*, *Gorilla gorilla*, *Homo sapiens* and *Pan troglodytes*; *Bos taurus*; *Sus scrofa*; *Canis familiaris*; *Equus caballus*. Similarly for flies, there are five groups composed of: *D.* *melanogaster*, *D. sechellia*, *D. simulans*, *D. yakuba* and *D. erecta*; *D.* *ananassae*; *D.* *pseudoobscura* and *D. persimilis*; *D.* *willistoni*; *D.* *grimshawi*, *D. mojavensis* and *D. virilis*.

A site instance must be found in at least three of these groups (with an allowed shift of up to 20 nt with the reference species) to be considered conserved by *Imogene*.

### Evolutionary models

*Imogene* can use two different evolutionary models, which vary in complexity and computational time, to compare orthologous binding sites. In both models, the bases within a site evolve independently of each other.

#### Felsenstein model

The simplest models of TFBS nucleotide evolution are copied on models of neutral evolution for genomic nucleotides. This procedure has been proposed by Sinha *et al*. (29,41) with the Felsenstein model of neutral evolution (42). In this TFBS evolution model, the transition probability from nucleotide *b* to *b*′ at position *i* in two sites at evolutionary distance *d* is defined as

(2) \begin{equation*} p_{b\rightarrow b^{\prime }}^i=q\ \delta _{b,b^{\prime }}+(1-q)\ w_{i,b^{\prime }}, \end{equation*}

where $\delta _{b,b^{\prime }}$ is the Kronecker symbol, $w_{i,b^{\prime }}$ is the mean frequency of base *b*′ at position *i* of the site (as given by the PWM model), and *q* is the probability of conservation for an evolutionary distance *d* under neutral selection (see below).

When two species are close to one another, *q* ∼ 1 and the probability that the observed bases are identical is high. On the contrary, when the two considered species are distant (*q* ∼ 0), the observed bases are uncorrelated and reflect the PWM probabilities $w$_*i*, *b*_.

The probability of conservation *q* can then be computed within this model by setting the PWM probabilities $w$_*i*, *b*_ to the mean genomic frequencies π_*b*_:

(3) \begin{equation*} q=\exp \left(-\frac{d}{1/2+4\pi _\textrm {A,T}\pi _\textrm {C,G}}\right), \end{equation*}

with $\pi _\textrm {A,T}$ (resp. $\pi _\textrm {C,G}$) the common genomic frequency of A and T (resp. C and G).

#### Halpern–Bruno model

The Halpern–Bruno model (HB) (43) differs in two ways from the simplest *Felsenstein* model. It uses the more complex Hasegawa, Kishino and Yano model (HKY) (44) for the neutral evolution of nucleotides and adds a fixation probability based on fitness differences for the evolution of nucleotides within the TFBS.

The HKY model improves on the Felsenstein model by taking into account the observed dependence of the mutation rate on the chemical nature of the bases. Substitutions between bases of the same chemical nature (purine or pyrimidine), also called transitions, are generally more frequent than the other type of mutations called transversions. This is encapsulated in the HKY model by the parameter *κ* which is the ratio of the transition rate over the transversion rate. It is measured to be *κ* = 2 in flies and *κ* = 3.7 in mammals (45).

Within a TFBS, the HB model extends the HKY model to take into account an additional purifying selection on the nucleotide identities (43). It is formulated by the following transition probabilities:

(4) \begin{equation*} p_{b\rightarrow b^{\prime }}=\exp (t\mathbf {H})_{b,b^{\prime }}, \end{equation*}

where ${H}$ is the rate matrix defined by

(5) \begin{equation*} H_{b,b^{\prime }} = \left\lbrace \begin{array}{@{}l@{\quad }l@{}}\pi _{b}\ h_{b^{\prime }\rightarrow b} & \text{if } b\ne b^{\prime }\\ - \sum _{b^{\prime }\ne b} H_{b,b^{\prime }} & \text{if } b = b^{\prime }. \end{array}\right. \end{equation*}

The evolutionary time *t* is expressed in terms of the evolutionary distance by

(6) \begin{equation*} t=\frac{d}{1/2+4\kappa \, \pi _\textrm {A,T}\pi _\textrm {C,G}}. \end{equation*}

Finally, the transition rates are defined by

(7) \begin{equation*} h_{b\rightarrow b^{\prime }}=\frac{w_{\mathrm b^{\prime }}}{\pi _{\mathrm b^{\prime }}}\ \frac{\log \left(\frac{\pi _{\mathrm b}w_{\mathrm b^{\prime }}}{\pi _{\mathrm b^{\prime }}w_{\mathrm b}} \right)}{w_{\mathrm b^{\prime }}/ \pi _{\mathrm b^{\prime }}- w_{\mathrm b}/ \pi _{\mathrm b}} \alpha _{b\rightarrow b^{\prime }} \end{equation*}

with $\alpha _{b\rightarrow b^{\prime }}=\kappa$ for a transition and $\alpha _{b\rightarrow b^{\prime }}=1$ for a transversion.

### Inference

The algorithm infers in a Bayesian way the PWM ${w}$ frequencies $w$_*i*, *b*_ based on observations of binding sites, as previously described in (14). In a Bayesian framework, the posterior distribution $\mathcal {P}({w}|\lbrace \mathcal {A}\rbrace )$ that the matrix ${w}$ represents the PWM binding to a set of aligned nucleotides $\lbrace \mathcal {A}\rbrace$ is proportional to the product of

- the *a priori* probability $\mathcal {P}_{{\rm ap}}({w})$, the ‘prior’, that the matrix $ {w}$ represents a PWM,
- the probability $\mathcal {P}(\lbrace \mathcal {A}\rbrace |{w})$ of observing the set of aligned nucleotides given that they belong to binding sites for the PWM ${w}$.

The prior is taken to be a Dirichlet distribution with parameters α_β_ at each PWM position,

(8) \begin{equation*} \mathcal {P}_{{\rm ap}}(w_{i})\ \propto \prod _{b \in \lbrace A,T,C,G\rbrace } w_{i,b}^{\alpha _b-1}. \end{equation*}

The nucleotides at different positions are assumed to be independent and the prior for the full site is taken to be the product of the $\mathcal {P}_{{\rm ap}}(w_{i})$ over the different positions. The parameters α_*b*_ are taken to be equal for Watson–Crick complementary nucleotides since a sequence and its reverse complement are not distinguished in the description of binding sites (i.e. we assume that binding is not biased toward a particular DNA strand). The two values of α_*b*_ are fully determined by assuming that (i) TFBS *a priori* have the same nucleotide frequencies as the background and (ii) that a PWM mean *a priori* information content is equal to the input threshold score *S*_*g*_.

The probability $\mathcal {P}(\lbrace \mathcal {A}\rbrace |{w})$ of observing the set of aligned nucleotides given the PWM ${w}$ is computed in a standard way (42) by recursion for a given PWM ${w}$ and a given evolutionary model.

The posterior distribution of the nucleotide frequencies at position *i* is thus obtained under the form

(9) \begin{equation*} \mathcal {P}(w_{i}|\lbrace \mathcal {A}\rbrace )\ \propto \prod _{a \in \lbrace \mathcal {A}\rbrace } \mathcal {P}(a|w_{i,b})\ \prod _{b \in \lbrace A,T,C,G\rbrace } w_{i,b}^{\alpha _b-1} \end{equation*}

where we omit the normalization factor.

In the idealistic case where the aligned nucleotides represent independent observations (infinitely distant species), the likelihood reduces to a multinomial distribution and the posterior is given by

(10) \begin{equation*} \mathcal {P}(w_{i}|\lbrace \mathcal {A}\rbrace )\ \propto \prod _{b \in \lbrace A,T,C,G\rbrace } w_{i,b}^{N_b + \alpha _b-1}, \end{equation*}

where *N*_*b*_ is the number of times the base *b* is observed in $\lbrace \mathcal {A}\rbrace$. This formula allows simple analytic formulations for the estimator of mean and maximum posterior probability. The mean posterior estimate is expressed as

(11) \begin{equation*} \tilde{w}_{i,b} = \frac{N_b+\alpha _b}{\sum _b N_b+\alpha _b}. \end{equation*}

Equation (11) coincides with the maximum likelihood estimate for a Dirichlet ‘prior’ with parameters α_*b*_ + 1.

In the case of a non-trivial evolutionary tree (like those of Supplementary Figure S1, the orthologous sites are correlated by their evolution from common ancestors. The probability $\mathcal {P}(a|w_{i,b})$ is a polynomial function of the $w$_*i*, *b*_'s. However, it generally lacks a simple analytical expression and the mean posterior estimate should be computed numerically.

### Mean posterior estimation

The mean posterior estimate was initially computed using a Markov chain Monte Carlo procedure (46). This turned out to be a time-consuming step in the algorithm. To speed it up, we observed, as noted above, that the mean posterior estimate for a prior with Dirichlet parameters α_*b*_ coincided with the maximum likelihood estimate for a prior Dirichlet parameter α_*b*_ + 1 in the case of uncorrelated observations as well as fully correlated ones (i.e. reducing to a single observation). We thus reasoned that maximization with this modified Dirichlet prior could give a quick satisfying approximation for the phylogenetic trees of Supplementary Figure S1, which was checked on different examples. This procedure is thus adopted in the present version of *Imogene* and for the results shown here. The posterior distribution obtained with the modified prior is maximized by using the Nelder–Mead simplex algorithm, as implemented in the GNU GSL. The initial value for the estimation is taken to be the mean estimator in the independent species regime given in Equation (10). This allows one to start close to the quadratic region and ensures fast convergence.

### A simple example of nucleotide inference using the two evolutionary models

To illustrate the inference of ancestral nucleotides and the main features of the two models, we consider in Supplementary Figure S2 a dinucleotidic genome with bases *X* and *Y* and a simple phylogenetic tree with an ancestral species at equal evolutionary distance from the reference species and a daughter species. We suppose that the observed nucleotide at position *i* of an observed binding site is *X*, both in the reference and the orthologous species.

Our goal is to infer the frequencies $w$_*Y*_ and $w$_*X*_ = 1 − $w$_*Y*_. First, there are two simple cases. For *d* = 0, the observations of the same nucleotide in the two evolutionary branches really constitute only one observation of *X*. On the contrary, for very long evolutionary branches *d* → ∞, the two instances of nucleotide *X* form two independent observations. Using the previous result (Equation (11)) with α_*X*_ = α_*Y*_ = α, the estimator of the maximum transformed posterior distribution for *N*_*X*_ and *N*_*y*_ independent instances of *X* and *Y* is

(12) \begin{equation*} w_Y = \frac{N_Y+\alpha }{N_Y+N_X+2\alpha }. \end{equation*}

Thus, for *d* = 0, the inferred frequency is

(13) \begin{equation*} w_Y = \frac{\alpha }{1+2\alpha } \end{equation*}

while for *d* → ∞, it tends toward

(14) \begin{equation*} w_Y = \frac{\alpha }{2+2\alpha }. \end{equation*}

Between these two extreme cases, an evolutionary model has to be used to estimate $w$_*Y*_ for finite evolutionary branches of length *d*.

For the Felsenstein model, the likelihood function writes

(15) \begin{equation*} \begin{aligned} \mathcal {P}(\mathcal {A}|w) & = w_X\,[q+(1-q)w_X]^2 + w_Y(1-q)^2w_X^2 \\ & = q^2w_X + (1- q^2) w_X^2 \end{aligned} \end{equation*}

where $\mathcal {A}$ stands for the simple alignment considered in Supplementary Figure S2 and we used $w$_*X*_ = 1 − $w$_*Y*_. From this expression it can clearly be seen that the evolutionary model simply interpolates between the independent species case (*d* → ∞, *q* = 0), where there are two observations of base *X*: $\mathcal {P}(w|\mathcal {A}) = w_X^2$, and the fully correlated case (*d* = 0, *q* = 1) where the two species merge and we have only one observation: $\mathcal {P}(w|\mathcal {A}) = w_X$. The corresponding mean $w$_*Y*,me_ and maximum posterior $w$_*Y*,ma_ analytic estimates for finite *d* read

\begin{equation*} \begin{split} w_{Y,{\rm me}}\ =\ &\frac{\alpha }{2} \, \frac{1+ q^2}{\alpha +1+\alpha q^2}\\ w_{Y,{\rm ma}}\ =\ &\frac{1}{4(\alpha +1)(1-q^2)} \bigg [ 3 \alpha +2 - (\alpha +1) q^2\\ &- \sqrt{[\alpha +2-3(\alpha +1)q^2]^2+ 8 q^2(1-q^2)(\alpha +1)^2}\bigg ]. \end{split} \end{equation*}

Note that for the maximum posterior estimate, $w$_*Y*,ma_, the prior exponent α + 1 has been used instead of α as explained above. So, the two estimates coincide at *q* = 0 and *q* = 1. Both estimates are plotted as function of the evolutionary distance *d* in Supplementary Figure S2 (α = 0.1).

For the HB model, the analogous results have been computed numerically and are also shown for comparison in Supplementary Figure S2. The HB model results are seen to be closer to the large distance limit than the Felsenstein model ones. Moreover, the difference between the nature of the estimates is seen to be comparable to the difference between the evolutionary models.

### Filtering of motifs coming from simple repeats

*Imogene* pre-processes the training set by masking repeated sequences with repeat masker (47) but this is not sufficient to eliminate the production of motifs corresponding to repeated sequences. These motifs have a non-Poissonian distribution of binding sites on intergenic sequences: one binding site has a high probability to be followed by another one after a multiple of the repeat period. This anomalous distribution of binding sites biases motif ranking and diminishes the algorithm CRM predicting power (14). Motifs corresponding to repeated sequences are thus filtered out using the non-Poissonian characteristics of their binding site distribution. The binding sites of each motif *m* are determined on the above-described set of *N*_bg_ = 10^4^ background sequences of length *L* = 2 × 10^3^ nt. For a Poisson distribution, one would expect the number $N^{(p)}_m(j)$ of intergenic sequences containing *j* binding sites to be

(16) \begin{equation*} N^{(p)}_m(j)=N_{{\rm bg}} \frac{(\lambda ^{({\rm bg})}_m L)^j}{j!}\, \exp (- \lambda ^{({\rm bg})}_m L), \end{equation*}

where $\lambda ^{({\rm bg})}_m$ is the computed density of binding sites of the motif *m* in the set of background sequences. The deviation from this theoretical Poisson distribution is quantitatively assessed by computing the χ^2^-like value,

(17) \begin{equation*} \chi ^2(m)= \sum _j \frac{[N_m(j)-N^{(p)}_m(j)]^2}{N^{(p)}_m(j)} \Theta (N_m(j)), \end{equation*}

where Θ is the Heaviside function (Θ(*x*) = 0 for *x* < 0, Θ(*x*) = 1 for *x* > 0) that restricts the sum to non-zero values of *N*_*m*_(*j*). Only the 75% motifs with the lowest χ^2^(*m*)-value are retained for subsequent computations.

### Distance between motifs

The similarity between two motifs is quantitatively assessed based on the overlap between the sets of their binding sites. The ‘strict proximity’ between motifs represented by two PWMs $\mathbf {w_1}$ and $\mathbf {w_2}$ is defined by

(18) \begin{eqnarray*} \mathrm{Prox(\mathbf {w}^{(1)},\mathbf {w}^{(2)})}=\nonumber \\ 2 \frac{\mathrm{Prob}\left\lbrace \left[S(\mathbf {s},\mathbf {w}^{(1)})>S_{{\rm th}}\right]\ \textrm {and}\ [S\left(\mathbf {s},\mathbf {w}^{(2)})> S_{{\rm th}}\right]\right\rbrace }{\mathrm{Prob}\lbrace S(\mathbf {s},\mathbf {w}^{(1)}) >S_{{\rm th}}\rbrace +\mathrm{Prob}\lbrace S(\mathbf {s},\mathbf {w}^{(2)})>S_{{\rm th}}\rbrace }, \end{eqnarray*}

where $\mathrm{Prob}\lbrace S(\mathbf {s},\mathbf {w})>S_{{\rm th}}\rbrace$ is the probability that a sequence $\mathbf {s}$ drawn at random with the background frequencies π_*b*_ has a binding score $S(\mathbf {s},\mathbf {w})$ (Equation (1)) above the threshold *S*_th_ for the frequency matrix $\mathbf {w}$. The strict proximity is computed analytically as explained in (14), where it was defined. To take into account potential shifts in the motifs or in their orientation, $\mathrm{Prox}(\mathbf {w}^{(1)},\mathbf {w}^{(2)})$ is computed for all possible alignments of the two matrices (with a maximum shift of *l*_*m*_/2 where *l*_*m*_ is motif size) in the two possible orientations. When shifted matrices are compared, they are completed by additional columns with the background frequencies (i.e. with no specificity). The proximity between two motifs is obtained simply by taking the maximum over the obtained strict proximities. It goes from 1 for two identical motifs to 0 for motifs that do not share any binding site above the threshold. *Imogene* distance between two motifs is defined as minus the logarithm of their proximity.

### Ranking motifs

The previous filtering step provides for each considered motif *m* the density $\lambda ^{({\rm bg})}_m$ of its binding sites on the background sequences and ensures that these sites are approximately distributed in a Poissonian way. The deviation from this baseline distribution on the CRM of the training set (t.s.) is used to score each motif. This is quantified by the Poissonian log-likelihood of the training set

(19) \begin{equation*} Pl(m)=\ \sum _{t\in \lbrace \mathrm{t.s.}\rbrace } \log \left(\frac{\left(L_t \lambda ^{({\rm bg})}_m\right)^{k_t} \exp (-L_t \lambda ^{({\rm bg})}_m)}{k_t!}\right), \end{equation*}

where *k*_*t*_ is the number of instances of *m* on the training set sequence *t* of length *L*_*t*_. Larger deviations from the baseline Poissonian distribution are supposed to reflect motif specificity for the training set and correspond to more negative/better scores.

### Scoring intergenic sequences

Given a list of motifs *m*_*i*_, a CRM *E* is scored as follows:

(20) \begin{equation*} S(E)=\sum _{i} n(E,m_i) \log (\lambda _i^t/\lambda _i^b), \end{equation*}

where *n*(*E*, *m*_*i*_) is the number of binding sites for the motif *m*_*i*_ on *E* and $\lambda _i^t$, $\lambda _i^b$ are the average number of binding sites per base on the training set and background respectively. It is important to note that the previously found motif binding sites are masked when scanning with successive motifs. Thus motifs with lower ranks that resemble high-ranking motifs do not increase artificially the CRM weight by predicting the same binding sequences twice.

### Selection of optimal intergenic sequences

When ranking genome-wide intergenic sequences, with a list of *N* motifs, the best intergenic sequence at a given position is determined as follows. The list of motifs is used to scan the genome for conserved binding sites above a given threshold. Binding sites are then grouped in successive CRMs of size *L* such as to maximize clustering. The position *E*_*i*_ of the center of the enhancer *i* is chosen to be the center of the motifs cluster:

(21) \begin{equation*} E_i = \frac{X_1+X_N+l_m-1}{2}, \end{equation*}

where *X*_1_ and *X*_*N*_ are the starting positions of the first and last TFBSs in the cluster and *l*_*m*_ is the width of the motif.

### Mammalian predictions

#### Learning sets, test sets and background test sets

For each class, the CRMs were divided into a learning set composed of 15 CRMs chosen at random, the other CRMs (∼20) defining the test set of ‘True Positives’. In addition, a set of background test regions was built using the 1 kb flanking sequences of the full list of CRMs.

Such an ‘adapted’ background test set was used to provide a more stringent and informative test of the algorithm. It prevents discrimination on the training set from the background test set based on other features than the sought high-information-content motifs such as a local composition bias. Furthermore, in order to avoid biasing the results toward the True Positives, uninformative sequences for *Imogene* (i.e. sequences where no binding site could possibly be found given *Imogene* conservation requirements) were also removed from this background test set. This yielded background test sets of 72 CRMs for the limb and 57 for the neural tube.

#### Cross-validation protocol

The learning set was used to learn the motif content. The 10 best motifs were then used to score test set CRMs and background regions. Because the length of the training set CRMs could vary, we decided to keep for each test sequence the best scoring 1 kb fragment. This process was repeated 40 times, and both generation and scanning threshold were varied. The retrieval rate of test set CRMs (True Positives) among background elements (False Positives) as a function of the score was used to build a Receiver Operating Characteristic (ROC) curve. The Area Under ROC Curve or AUC, a quantity that varies between 0 for absolute misclassification, 0.5 for random classification, and 1 for perfect classification, was used to evaluate the quality of prediction. The parameter set yielding the highest AUC was chosen as the best set.

### Comparison with Kantorovitz *et al.*

The data provided by Kantorovitz *et al.* (22) consisted of eight classes of human CRMs, each class containing between 10 and 67 CRMs, with an average of 30 CRMs per class. Coordinates of the human CRMs were obtained from http://enhancer.lbl.gov and were converted from the human hg19 to the mouse mm9 assembly using the UCSC LiftOver tool, yielding a loss of 5–10 unmapped CRMs per class. The extraction of the mammalian alignments with Imogene resulted in another loss of 1–2 CRMs per class for which no alignment could be retrieved. Overall, the ratio of finally retrieved over initially available number of CRMs per class was the following: dorsal root ganglion (7/10), eye (12/16), forebrain (50/67), heart (9/19), hindbrain rhombencephalon (25/32), limb (24/35), midbrain mesencephalon (36/42) and neural tube (20/23). The sensitivity of Imogene on these CRMs was then computed using a leave-one-out cross-validation scheme as described in (22). More precisely, we measured the ability of Imogene to retrieve a bona fide CRM of a given class (the test CRM) embedded in 10 kb of background intergenic DNA using the best motifs generated from the other CRMs of the class (the training set). The sequence containing the test CRM and the intergenic DNA was scanned using a window size equal to the average length of the CRMs in the given class. The window of highest score defined the predicted CRM. The prediction was considered valid when the test CRM and the predicted CRM overlapped over at least 100 bp. The sensitivity was finally computed as the proportion of test CRMs retrieved. This process was repeated using 10 different background regions. Imogene parameters were varied (evolutionary models: F and HB, *S*_*g*_: 11 and 13, *S*_*s*_: 5–13, number of motifs: 1–15), and the parameters giving the highest mean sensitivity over the 10 cross-validations were kept for each class. A *p*-value of 0.05 was computed empirically as described in (22). More precisely, the above process was repeated by drawing at random the best scoring fragment. The proportion of correctly predicted CRMs was computed. The process was repeated 100 000 times. The threshold sensitivity expected by chance with a *p*-value of 0.05 was obtained as the threshold proportion above which the 5000 higher computed proportions lied.

Two separate sets of tests were performed, either with or without making use of multiple sequenced genomes and conservation at the CRM retrieval step. Without conservation, tests were performed on human CRMs embedded in human intergenic background using the exact same composite sequences as in (22). For *Scangen* in its normal mode with conservation, the mouse is the reference species and alignments for the background sequences used in (22) needed to be retrieved. To do so, we followed the same protocol as for the training set sequences using the UCSC LiftOver tool for coordinate conversion and extracting alignments with Imogene. Because the length of the final background sequences could change during the process, we redefined 10 kb background regions around the centers of the sequences in the mouse genome. The training set alignments were then embedded in the center of the corresponding 10 kb background alignments and repeats were masked using repeatmasker (47). The resulting sequences were finally used to conduct the leave-one-out cross-validation (LOOCV). We note that repeat masking did not significantly affect the results (data not shown).

### Leave-one-out cross-validation for the CRM discrimination task

Let us note $\mathcal {C}_i$ the tissue class of interest. There are *M*_*i*_ corresponding CRMs. Let *N*_*c*_ denote the total number of classes. Our goal is to find the particular motif signature that distinguishes these *M*_*i*_ CRMs from the *N*_*c*_ − 1 other classes of CRMs. This signature corresponds in our case to a number *N* of top ranked motifs with generation and scanning thresholds *S*_*g*_ and *S*_*s*_. These are the three parameters we wish to constrain with a LOOCV procedure.

Let us detail this procedure in the case where we distinguish class $\mathcal {C}_i$ from the other classes $\mathcal {C}_j$. The *M*_*i*_ CRMs of $\mathcal {C}_i$ are termed ‘positive’ CRMs and the *M*_*j*_ CRMs of each of the other classes are termed ‘negative’ CRMs. Let us note *M* = ∑_*i*_*M*_*i*_ is the total number of CRMs. The LOOCV consists in withdrawing one ‘test’ CRM from these *M* CRMs, learn the motifs on the *M* − 1 resulting CRMs, and use them to score the let alone test CRM. For the learning step, motifs are generated with threshold *S*_*g*_ on each class (one class being deprived of one CRM), yielding *N*_*c*_ sets of motifs: one set of positive motifs from class $\mathcal {C}_i$, and *N*_*c*_ − 1 sets of negative motifs from the other classes. The *N* top ranked motifs from each set are then used to scan the *M* CRMs for conserved instances with scanning threshold *S*_*s*_. Each CRM *E* is scored with respect to these *N*_*c*_ sets of motifs by

(22) \begin{equation*} S(E) = \sum _{j=1}^{N_c} (2\delta _{j,i} - 1) S_N^{\mathcal {C}_j}(E), \end{equation*}

where $S_N^{\mathcal {C}_j}(E)$ is the CRM score for the *N* top motifs of class $\mathcal {C}_j$ as defined below in the ‘Main program’ description, and δ_*j*, *i*_ = 1 if *j* = *i*, and 0 otherwise. This score simply gives positive contributions if positive motifs are found on the CRM, and negative contributions if negative motifs are found. This scoring procedure allows to rank the test CRM among the other *M* − 1 CRMs. Ties are resolved by attributing their mean rank to equally scored CRMs. The rank of the test CRM is used rather than its raw score to avoid potential bias stemming from score normalization. Indeed, the raw score is dependent on the generated motifs, which differ at each step of the LOOCV. This procedure is repeated over all *M* CRMs, yielding a corresponding list of *M* ranks. This list is finally used to build a ROC curve discriminating True Positives (CRMs from class $\mathcal {C}_i$) from False Positives (the other CRMs). The discrimination is quantified by the AUC for a false positive rate FPR ≤ 20%, which we notify as AUC20 and want to maximize.

In our case, we used a 2D parameter grid with *S*_*g*_ varying between 7 and 13 bits by steps of 1, and *S*_*s*_ varying between *S*_*g*_ − 5 and *S*_*g*_ by steps of 1. Both Felsenstein and HB models were used for motif generation. For each parameter set, the number of motifs used for scanning was increased from 1 to a maximum number of 10 (actually never attained) until the addition of a new motif decreased the AUC20, yielding an optimal number of motifs *N*. Finally, for each class, the parameter set {*S*_*g*_, *S*_*s*_, *N*} yielding the highest AUC20 was selected as the best parameter set.

### Motifs identification

In order to identify the known TFs that might correspond to the *de novo* generated motifs, we used the TRANSFAC (48) and JASPAR (49) databases, as well as the list of motifs provided in (50) from HT-Selex experiments and the UniPROBE database (51) created from protein binding array data.

In order to avoid uninformative matches, we kept motifs that had an information content greater than 8 bits, a threshold approximately corresponding to four conserved nucleotides. This led us to keep 764 vertebrate motifs (934 total minus 170 below threshold) in the TRANSFAC database, 389 vertebrate motifs (476 total minus 87 below threshold) in the JASPAR database 575 HT-Selex motifs (580 total minus 5 below threshold) and 488 in the UniPROBE database (538 total minus 50 below threshold).

Each *de novo* motif was compared to all kept motifs in each database, using the PWM distance introduced in (14). During the comparison, motifs are shifted to find the best match with a minimal match of 5 nt. The shift is simply introduced by adding flanking nucleotides with background frequency on either side. The closest candidate was kept for identification.

## RESULTS

### Description of Imogene

**Figure 1.**
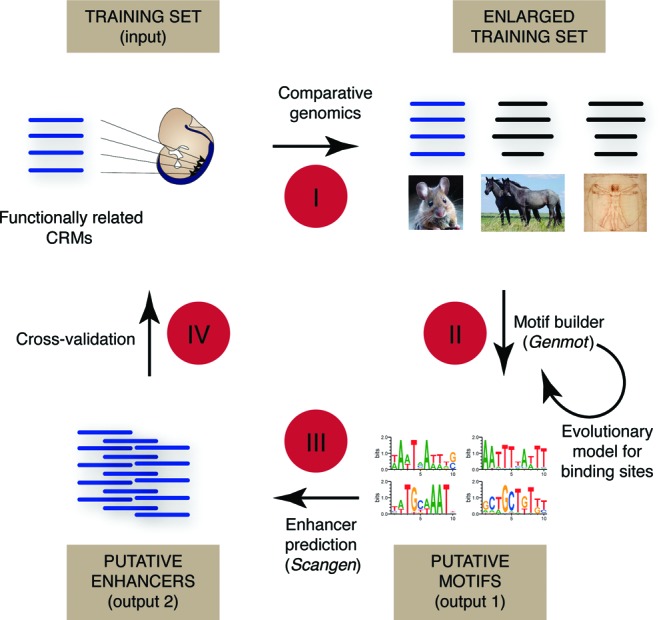
Imogene workflow**.** The algorithm takes as input a list of functionally related CRMs. Homologous sequences from closely related species are automatically retrieved (**I**) and scanned in order to generate a list of putative transcription factor motifs (**II**). These motifs fuel the last step consisting in the inference of related novel CRMs (**III**). These predicted CRMs can finally be compared to a set of test CRMs to evaluate the predictability power of the whole procedure (**IV**).

*Imogene* has two modes that can be used in succession, as shown in Figure 1 and summarized here (see the ‘Materials and Methods’ section for details of their implementation).

The first mode, *Genmot*, aims at extracting statistically meaningful PWMs from a ‘training set’ of functionally related CRMs on a reference genome (the mouse *M. musculus* genome for mammals; the *D. melanogaster* genome for flies). The cumulated size of the training set could in principle be unlimited, but in practice computer execution time requires it to stay below 100 kb. It should also be above a few kb to provide a sufficient amount of information (a training set of about 20 kb appears as a good compromise). Starting from a chosen training set, *Genmot* performs its task in two steps (I and II in Figure 1): (I) *Genmot* first enlarges the training set with aligned orthologous sequences in other related sequenced genomes (see ‘Genome alignments’ in the Materials and Methods section), as shown in Supplementary Figure S1 (for the mouse, the 11 other aligned mammalian sequenced genomes with high coverage presently available on the Ensembl project (15), the 11 other *Drosophilae* sequenced genomes (16) for the fly). This comparative genomics step results in the creation of the ‘enlarged training set’ (step I in Figure 1). (II) In this second central step, *Genmot* builds PWMs of a given length ℓ (10 nt is the default value) by scanning the training set in an iterative manner (step II in Figure 1). Each sequence of ℓ nucleotides in the training set is used in turn to create an initial PWM using a Bayesian prior. This PWM is then refined by scanning the training set to find all the PWM binding sites in the training set, i.e. all ℓ nucleotide long sequences in the training set that have a binding score above a generation threshold score *S*_*g*_ chosen at the procedure onset (*S*_*g*_ = 13 bits is the default value). These binding sites are filtered using conservation, i.e. only sites that have orthologs in distant species are further considered (see ‘Conservation requirements for binding sites’ in the Materials and Methods section). A shift in alignment between a binding site on the reference species and its orthologs in other species is allowed for the correction of eventual alignment errors (20 nt is the shift default value). The ensemble of conserved binding sites and their orthologs serve, using an evolutionary model, to build a refined PWM. The procedure is then iterated by finding the binding sites of the refined PWM and using them to build a further refined PWM until convergence to a stable set of binding sites.

The need of an evolutionary model to properly assemble binding sites (28,29,41) is simply explained. A binding site in the reference genome and its orthologs are all related through descent from their last common ancestor, and cannot therefore be considered as independent observations. In order to correctly quantify the amount of information provided by the observation of orthologous sites, one has to estimate their potential of change through mutation since their last common ancestor. To account for this, *Imogene* can, in its present implementation, make use of either one of two evolutionary models of TFBS evolution at the user choice. The first option, ‘Felsenstein model’, is a simple and computationally fast model proposed in (41). Mutations are generated at the same rate in a PWM binding site than in the background intergenic sequences. However, the mutated nucleotide in a binding site is drawn according to its frequency in the PWM at the mutated position. This is analogous to the simplest model of DNA evolution (42) but with nucleotide neutral relative abundances replaced by PWM nucleotide frequencies. The Felsenstein model is the simplest model that provides at evolutionary equilibrium nucleotide frequencies that agree with those prescribed by the PWM at the different positions in the binding site. The second option (43), ‘HB model’, uses an evolutionary model that is more complex than the Felsenstein model but is also more clearly grounded on theoretical population genetics ideas. It has previously been used for TFBS evolution in (28). It allows for the inclusion of different mutational probabilities between different bases in the neutral background intergenic mutation model. Additionally, it includes a fitness-dependent fixation probability for a mutation in a TFBS based on classical population genetics estimates for the fixation of a mutant allele appearing in a homogeneous population (52). The relative fitnesses of different nucleotides are determined by the requirement that binding site convergence to evolutionary equilibrium leads to the PWM nucleotide frequencies (see the Materials and Methods section for details).

The described procedure produces a PWM for each ℓ nucleotide long sequences in the training set. In a series of final steps (see the Materials and Methods section for a mathematically detailed description), this long list is pruned and ranked based on comparison of the PWM bindings sites on the training set to a ‘background’ set of intergenic sequences in the reference genome (20 Mb of *M. Musculus* or *D. melanogaster* genomic DNA). *Imogene* pre-processes the training set by masking repeated sequences with repeat masker (47) but, as noted in (14), this is not sufficient to eliminate some PWMs corresponding to repeated sequences from the produced list of PWMs. These PWMs have statistically anomalous distributions of binding sites that bias their subsequent ranking. Therefore, in a filtering first step, PWMs corresponding to repeated sequences are discarded on the basis of their anomalous distribution of their binding sites in the background set (see ‘Filtering of motifs coming from simple repeats’ in the Materials and Methods section). Then for each remaining PWM, the distribution of its conserved binding sequences on the training set is compared to the distribution of the PWM conserved binding sequences on the set of background intergenic sequences. The larger the statistical deviation between the two distributions, the larger the PWM score and the more meaningful the PWM is deemed (see ‘Ranking motifs’ in the Materials and Methods section). In a final step, PWMs in the ranked list are compared (see ‘Distance between motifs’ in the Materials and Methods section) and, among similar ones, only the highest scoring one is kept. Although the identity of the TFs corresponding to the different PWMs of interest is not directly assessable by the algorithm, the comparison between the produced PWMs and existing databases can provide relevant information on their identity, as will be shown in the following sections.

In its second mode, *Scangen*, *Imogene* determines intergenic sequences in the reference genome that are considered as putative CRMs with the same functional specificity as the training set. This second mode (step III in Figure 1) is based on the PWMs inferred in the *Genmot* mode. The algorithm scans the entire non-coding repeat-masked reference genome and finds all the conserved binding sites above the scanning binding score *S*_*s*_ for the N first PWMs in the ranked list. The intergenic sequences of a given length (the default value is 1000 nt) are then scored according to their similarity to the training set in their content of PWM binding sites (see ‘Scoring intergenic sequences’ in the Materials and Methods section). The closest the similarity in its motif content with the training set, the most likely an intergenic sequence is deemed to be functionally related to the training set.

### Application to mammalian developmental programs

In order to assess *Imogene* performance on mammalian transcriptional regulation, we applied it to two sets of mammalian specific CRMs that have previously been identified starting from p300 Chip-seq data and functionally tested in a transient transgenic assay for activity in stage 10 mouse embryo (11). We chose CRMs active in neural tube and limb, as characterized in the VISTA website (http://enhancer.lbl.gov). For each developmental program, a subset of CRMs was visually selected for specificity and strength of expression in the tissue of interest from the provided expression pattern. Among these selected sets, two limb CRMs and four neural tube CRMs contained no sequence that could possibly be used to learn motifs by *Imogene*, due to its conservation requirements, either because of repeat masking or because of low conservation (see the Materials and Methods section). Elimination of these uninformative sequences produced curated training sets of 29 neural and 39 limb CRMs (see ‘Training sets’ in the Materials and Methods section).

A cross-validation scheme was then used to measure *Imogene* predictability power (see ‘Materials and Methods’ for details). In brief, for each developmental program, the CRMs of the training set were divided into a learning set composed of 15 CRMs chosen at random, and a test set composed of the other CRMs used as True Positives.

**Figure 2.**
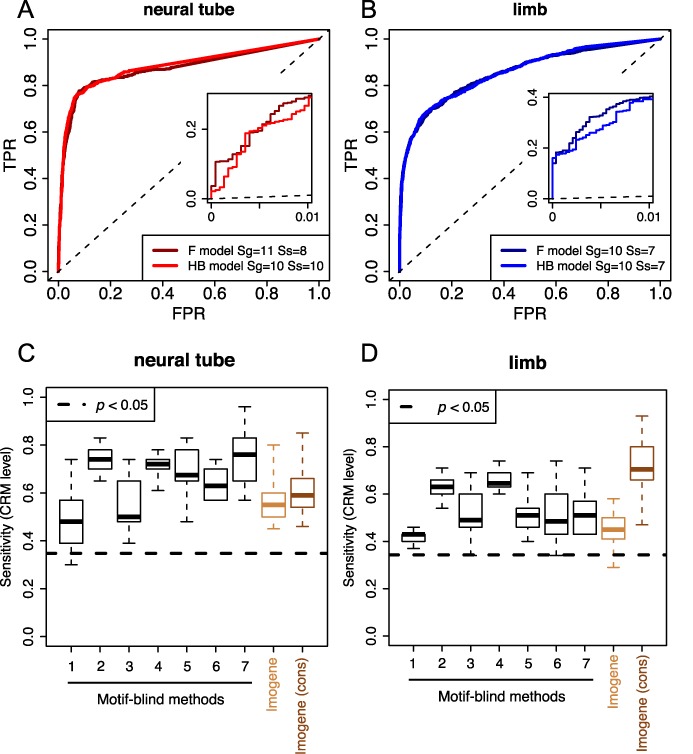
Analysis of well characterized developmental processes. We tested the algorithm on mammalian CRMs driving expression at E11.5 in neural tube (**A**) and limb (**B**). For each class, CRMs were divided into a training set and a test set. Motifs were learned on the training set and used to score CRMs from the test set along with background regions consisting of the CRMs' 1 kb flanking sequences (see the Materials and Methods section). The displayed ROC curves show the proportion of test set CRMs recovered above a given score (True Positive Rate denoted by TPR) versus the proportion of recovered background sequence at the same score for the Felsenstein (F) and Halpern–Bruno (HB) models. The shown ROC plots are the results of 40 trials. The FPR ≤ 1% region of each curve is replotted in the insets for better visibility. For each test set and each evolutionary model, the thresholds *S*_*g*_ and *S*_*s*_ used for motif generation and sequence scanning are given in the figures. Black dashed lines show random discrimination. (**C** and **D**) Imogene ability to predict neural tube (C) and limb (D) CRMs is compared to that of the ‘motif-blind’ methods assessed in (22). A leave-one-out cross-validation scheme is used to assess the efficiency of the different methods to predict a bona fide CRM embedded in a 10 kb intergenic region, as described in (22) and in the Materials and Methods section. The box plot summarizes the distributions of sensitivities obtained using 10 different intergenic regions. The dashed line indicates the sensitivity value above which results have a probability smaller than 5% to be generated by chance. The results obtained with *Imogene* are shown when only sequences from a single species (human) is considered at the prediction stage (light brown) or when conservation between mammalian genomes is also used (dark brown). The different methods examined in (22) are denoted by numbers in the figure with the following correspondence with the terminology of (22): 1 = HexDiff.rc, 2 = PAC.rc, 3 = HexYMF.s200.rc, 4 = HexMCD, 5 = D2z.cond.s100, 6 = D2z.cond.mo1.weights.rc and 7 = D2z.cond.weights. The HexDiff and HexYMF methods consider the words most associated in a statistical sense with the training set, and then use a weighted sum of word counts to score a given sequence. The PAC method (for Poisson Additive Conditional) also considers the words that are most associated with the training set, and then examines how over-represented each of these words is in the test sequence, relative to the assumed background, by computing a Poissonian *p*-value. The HexMCD method trains separate fifth-order Markov chains on training modules and background sequences, and quantifies which model matches the test sequence better using a score proposed in (65). The three different variants of the D2z method compute dot products between the k-mer frequency distributions of training and test set sequences, and use their statistical significance (*z* score) as a scoring scheme. A fuller description of each method is provided in (22).

The learning set was used for motif generation by *Imogene* in its *Genmot* mode. This procedure was conducted for both evolutionary models using different values of the generation parameter *S*_*g*_ and scanning threshold *S*_*s*_ to obtain the optimal values of these parameters for each model and each learning set (see Figure 2 and Supplementary Figure S3).

The test CRMs of the training set were then ranked, using motifs generated on the learning set, against a ‘background test set’, a set of ∼60 regions of 1 kb taken from the flanking sequences of the initial set of CRMs (see the Materials and Methods section).

For different parameter sets, the test CRMs as well as the intergenic sequences of the background set were scored. The proportion of retrieved test set CRMs above a given score (True Positive Rate or TPR) was plotted against the proportion of appearing test background regions above the same score (FPR) as this score decreased to produce a so-called ROC curve (53). The ROC curves corresponding to different parameter values were then compared using the AUC, a quantity that is maximal at best prediction. Supplementary Figure S3 shows the AUC as a function of the number of motifs *N* for different values of the scanning threshold *S*_*s*_. One can see that the AUC increases quickly with the first five motifs generated, and has nearly converged to its maximum value when 10 motifs are kept. Therefore, we restricted ourselves to *N* = 10 motifs, and constrained the other parameters using AUC maximization. Figure 2 shows the ROC curves obtained for the optimal parameters. They are seen to be similar for both models and both training sets. For the neural tube CRMs, 30% of the test set CRMs are retrieved at 1% FPR whereas an even larger proportion of 40% is obtained for the limb CRMs. The HB and the Felsenstein models are seen in Figure 2 to yield very similar results in both cases. This standard procedure provides a test of the two modes of *Imogene*. Its success indicates that meaningful motifs were generated at the *Genmot* stage and that they were properly used at the *Scangen* stage to recognize the test CRM from background sequences.

It should be noted that the test really provides only a lower estimate of *Imogene* success rate. Sequences of the background test set counted as ‘False Positive’ could, in reality, be bona fide positive CRMs.

One interesting feature of *Imogene* lies in its production of specific motifs. In our cross-validation procedure, different ranked lists of motifs were created for each randomly drawn test set. In order to provide a list of motifs generated by the algorithm, we ran *Imogene* on the full set of CRMs for each class. The corresponding 10 best motifs are shown in Figure S4. Figure S4 also shows the closest PWM to each motif in the TRANSFAC (48), JASPAR (49), HT-Selex (50) and UniPROBE (51) list of motifs, as computed by *Imogene* PWM distance. Previously characterized motifs belonging to the considered developmental programs appear in each class (e.g. Oct/Pou TF family and NeuroD motif in the neural CRMs). The motif content of each CRM is also provided in Supplementary Figures S5 and S6. It is seen that the 10 best motifs appear on most CRMs of the training set.

Among the existing algorithms, Kantorovitz *et al.* (22) concluded that ‘motif-blind’ methods were the most successful for characterizing the specificity of a small set of CRMs. In order to further assess *Imogene*, we thus chose to compare its performance to that of these algorithms. Kantorovitz *et al.* (22) benchmarked the prediction of the algorithms they examined on eight mammalian CRM datasets promoting expression in diverse tissues. We quantified the ability of *Imogene* to characterize these different sets of CRMs using the cross-validation protocol of (22) in which the CRMs to be tested were compared to intergenic sequences with similar GC content (see the Materials and Methods section). Conservation and phylogeny were used for the generation of motifs (i.e. in *Imogene Genmot* mode). The CRM scoring was performed both using conservation, the normal *Scangen* operating mode, and without it in order to provide a fair comparison between *Imogene* and the ‘motif-blind’ methods that do not take advantage of conservation. The results are displayed in Figure 2 for the neural tube and limb CRM datasets and in Supplementary Figure S7 for the other CRM datasets. In all cases, *Imogene* is found to perform comparably to the ‘motif-blind’ methods (22). Without conservation, it appears nonetheless less efficient than the best ‘motif-blind’ methods, such as ‘HexMCD’. The use of conservation significantly enhances *Imogene* predicting power and actually makes it the top predictor for several datasets (5/8). The prediction of specific motifs is, of course, the interesting complementary feature of *Imogene* in both cases.

### Discrimination of tissue-specific CRMs in the mouse

Given the ability of *Imogene* to distinguish specific CRMs from background sequences, we found it interesting to apply it to the related but distinct task of distinguishing different classes of CRMs. The question was previously considered for *D. melanogaster* CRMs based on ChIP-seq data at different developmental time points (38), as detailed in the next section. It consists in learning features that distinguish the CRMs of a given class from the CRMs of other classes in order to be able to predict the class of a newly observed CRM. The task differs from distinguishing CRMs from background intergenic sequences since motifs shared among different classes that , for instance, characterize the binding of generic CRM factors, are of no use for discrimination purposes.As a test case, we considered the neural tube and limb sets of mammalian CRMs used in the previous section. Given the nature of the task, we selected in each set the CRMs with an expression that appeared mostly restricted to neural tube and limb. This yielded 12 neural and 15 limb CRMs.

As in (38), we used a LOOCV scheme in which the learning set constituted all but one of the elements of a class, the remaining one being used as a test sequence. The process can be summarized as follows. We call the class of interest the positive class and the classes against which we wish to learn the negative classes. The LOOCV process begins with the exclusion of a (positive or negative) CRM that serves as an unobserved test CRM. Then, a set of *N* motifs is learnt on the remaining CRMs of each class, yielding positive and negative motifs. These motifs are used to build a simple linear classifier based on a weighted score giving positive (resp. negative) contributions to positive (resp. negative) motifs (see the Materials and Methods section). Finally, the test CRM is ranked among all CRMs by the build classifier and this rank is registered. A successful classification would rank positive CRMs on top of the list and attribute worse ranks to negative CRMs. Therefore, after processing all CRMs, the list of ranks for the positive and negative CRMs is represented as a ROC curve indicating the TPR and FPR for increasing rank. This serves to optimize the different parameters (the threshold for motif generation *S*_*g*_, the threshold for sequences scanning *S*_*s*_, and the number of motifs *N* used to score sequences) by maximizing the AUC for a FPR ≤ 0.2.

**Figure 3.**
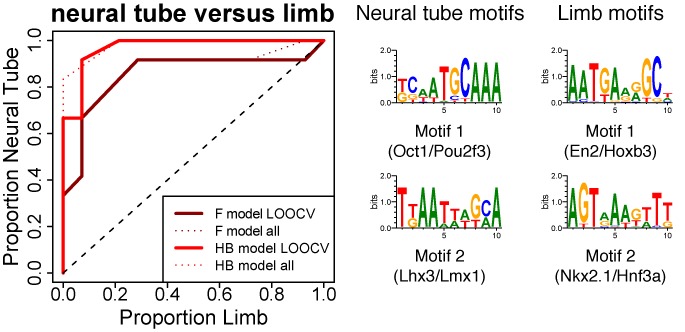
Pattern recognition (mammals)**.** ROC plots showing the discrimination between limb and neural CRMs using a simple linear classifier. Neural and limb classes are compared to each other. Thick lines correspond to a leave-one-out cross-validation (LOOCV) scheme with a score function based on the *de novo* generated motifs from *Imogene*. The results obtained with the two evolutionary models are shown (Felsenstein model (F), solid dark red line, with threshold parameters *S*_*g*_ = 11, *S*_*s*_ = 9; Halpern–Bruno (HB) model, solid light red line, with threshold parameters *S*_*g*_ = 11, *S*_*s*_ = 8). The analogous discrimination curves based on learning motifs on the whole training set (with the same threshold parameters) are shown for comparison (colored dashed lines). With this latter procedure, the discrimination is improved but is still comparable to that computed by the LOOCV, indicative of no strong overfitting of the training set. The corresponding discriminative motifs are shown for the whole training set learning with HB model (similar motifs are obtained with the F model). Black dashed line shows random discrimination.

The results are shown in Figure 3. We focus on the results obtained with the HB evolutionary model. Results (motifs and thresholds) are very comparable with the Felsenstein model. Motifs are shown on the right of the ROC plots and were generated on the positive classes with optimal parameters. The two classes were optimally discriminated using only two motifs in each class, with specificities *S*_*g*_ = 11, *S*_*s*_ = 8, comparable to that found in the learning task of the previous section. The closest motifs in the TRANSFAC (48) and JASPAR databases (49), as well as in the HT-Selex experiment list (50) and UniPROBE database (51), are shown in Supplementary Figure S8. The best ranking motif of the neural CRMs is found to be unequivocally associated with the TRANSFAC Oct/Pou Transcription Factor known to be involved in the neural tube formation (54).

### Discrimination of *Drosophila* tissue-specific CRMs

In order to further test the discriminating power of *Imogene de novo* generated motifs, we applied it to the CRM classification task reported in (38). In this work, previously characterized *D. melanogaster* CRMs were divided into five classes corresponding to the different tissue types in which they were active: mesoderm (meso), somatic muscle (SM), visceral muscle (VM), mesoderm and somatic muscle (meso & SM) and visceral and somatic muscle (VM & SM). Zinzen *et al.* (38) made use of a collection of Chip-seq binding data for different factors and at different developmental time points to attribute to each CRM a total of 15 peak height values. It was then tested whether classical machine learning techniques could be used to discriminate the different CRM classes on the basis of these extensive data. This was indeed found possible with a high success rate in a standard cross-validation scheme: CRMs predicted to belong to a given class with a probability higher than 95% were indeed found to belong to that class with a high success rate of 80%.

This led us to wonder whether *Imogene* would succeed in classifying these different CRMs without using any binding data, but rather on the basis of combinations of *de novo* motifs that it would itself generate. We used the set of well-characterized CRMs belonging to five different classes assembled in (38). We then proceeded as in the previous case of mammalian CRMs.

**Figure 4.**
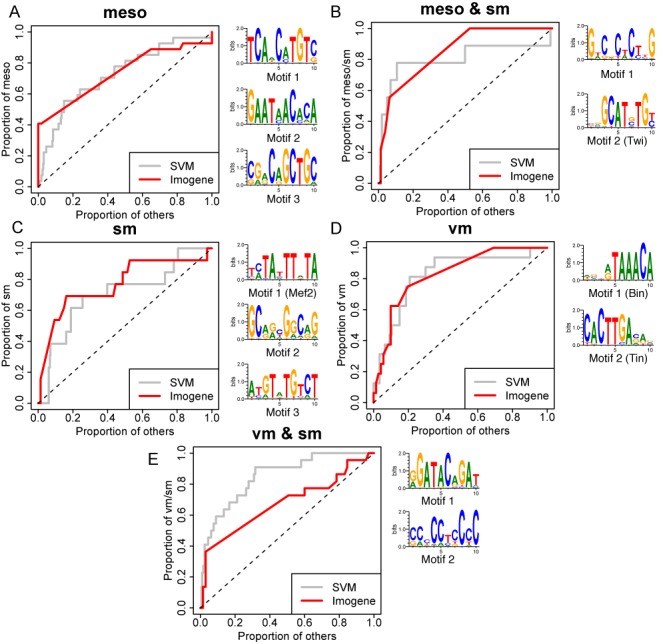
Pattern recognition (*Drosophilae*). Recognition of classes of CRMs expressed in five tissue types: mesoderm (meso), somatic muscle (sm), visceral muscle (vm) , mesoderm and somatic muscle (meso & sm) and visceral and somatic muscle (vm & sm). ROC plots are obtained using a leave-one-out cross-validation scheme. Two classifiers are compared: a Support Vector Machine using 15 ChIP-on-chip peak heights (gray, replotted using the data and the program provided in (38)), and *Imogene* using the *de novo* generated motifs with Felsenstein evolutionary model (red) and a simple linear classifier (see the Materials and Methods section). The following thresholds were used: meso (*S*_*g*_ = 12, *S*_*s*_ = 12), meso & sm (*S*_*g*_ = 10, *S*_*s*_ = 10), sm (*S*_*g*_ = 9, *S*_*s*_ = 4), vm (*S*_*g*_ = 10, *S*_*s*_ = 10) and vm & sm (*S*_*g*_ = 11, *S*_*s*_ = 8).

*Imogene* results are shown together with the machine learning results of (38) in Figure 4. For clarity, we here show results obtained with the Felsenstein model. Results obtained with the HB model are comparable. Strikingly, without any binding data, *Imogene* prediction rates are comparable to the machine learning ones in the specificity range (FPR ≤ 5%) used for CRM prediction in (38). Its performance is even better for the Meso and SM classes at high score. The latter case is of particular interest. The machine learning algorithm essentially used Mef2 ChIP-seq peak heights to predict SM CRMs, resulting in an incorrect classification at high scores since this TF is required for the differentiation of all muscle types. However, the use of the specific Mef2 motif obtained *de novo* from the SM training set allows one to restore a correct classification at high score (Figure 4C).

On the side of each ROC plot, the *de novo* motifs generated on the whole training set are displayed. The number of motifs shown is the optimal number used for CRM scoring in the leave-one-out cross-validation. Among the generated motifs, one can recognize 4/5 TFs for which ChIP-seq data were used in (38), namely Twist (motif 2, meso & SM), Mef2 (motif 1, SM), Bin and Tin (motifs 1 and 2, VM). The Bap motif was not found by the algorithm and correspondingly it was not shown to be of importance in (38).

In summary, our analysis indicates that *Imogene* not only determines *de novo* functionally relevant binding sites within a set of CRMs but can also be used to identify the more subtle differences in binding sites that underlie functional differences between related sets of CRMs.

### Web interface

**Figure 5.**
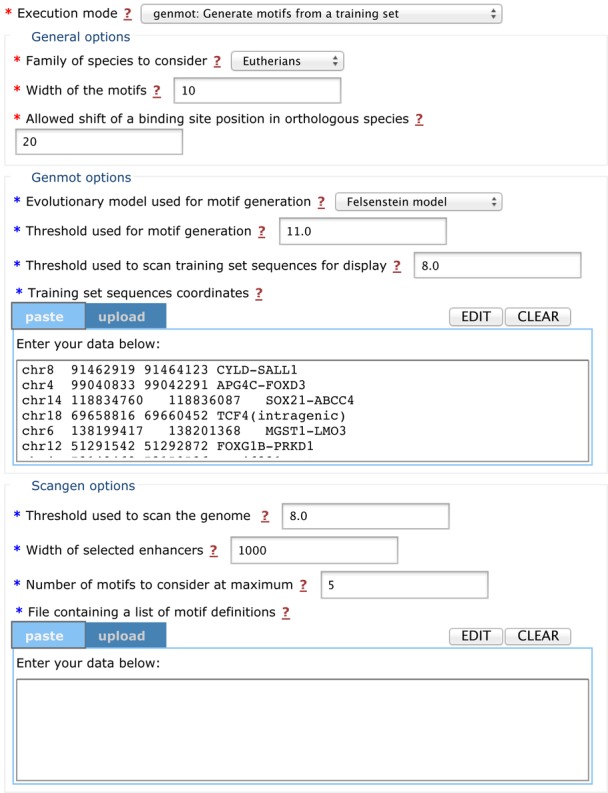
Web-based interface: input web page. A copy input web page for *Imogene* powered by the mobyle bioinformatics framework is shown.

**Figure 6.**
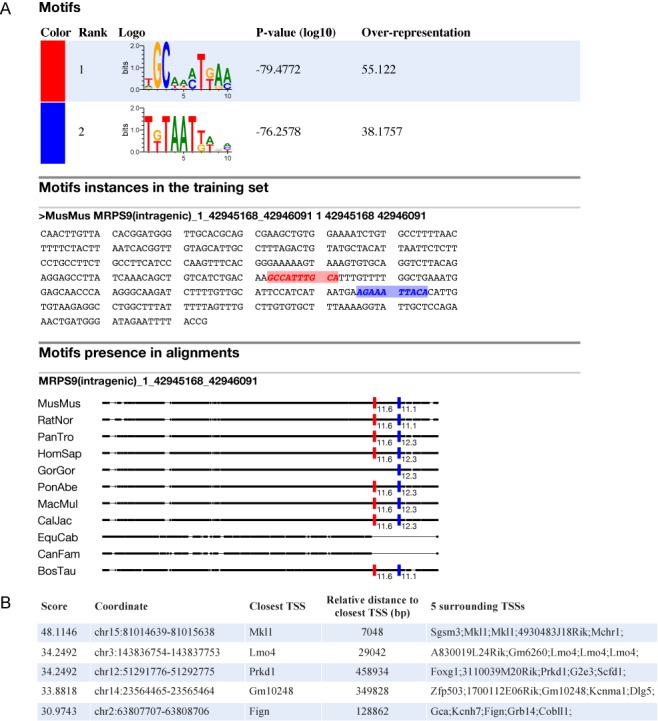
Web-based interface: output web page. Example of an output web page for *Imogene* powered by the mobyle bioinformatics framework. (**A**) Result page for the *Genmot* mode. Two motifs were generated from the neural tube full training set (default is five) using the same parameters as in Figure 2. Results are shown for the training set sequence MRPS9 (intragenic). For display purposes, the beginning of the sequence, which contains no instances for the motifs, was cut in the middle panel. In the alignments, thick lines correspond to sequences and thin lines to gaps. (**B**) Result page for the *Scangen* mode. The two generated motifs were used to score putative regulatory sequences of 1 kb in the mouse genome at optimal threshold *S*_*s*_ = 10. The five best ranking sequences are shown (default is 200).

The ensemble of developed statistical tools and the allied computer codes are freely available at http://github.com/hrouault/Imogene. In addition, they can be used through a user-friendly web interface (http://mobyle.pasteur.fr/cgi-bin/portal.py#forms::imogene) that provides motif and CRM predictions for the community. This interface is powered by the Pasteur Institute Internet server through the mobyle framework (55). The input web page and an example output web page are shown in Figures 5 and 6 respectively.

The input form (see Figure 5) is divided into several sections. One of the two available algorithm modes should be chosen at start:

- Genmot: given a list of coordinates of typically 15 enhancers of 1 kb (training set), generates *de novo* motifs ranked by their score (*Pl*(*m*) in the Materials and Methods section).
- Scangen: given the previously generated motifs, produces a list of genome-wide predicted CRMs with conserved binding sites. The rank of a CRM is based on a Poissonian score that takes into account the CRM content in motifs (as described in the Materials and Methods section)

The group of species considered should also be specified. The algorithm can be used on *Drosophilae* (with reference species *D. melanogaster*) or mammals (with reference species *M. musculus*). The different algorithm parameters such as the sought motif width, threshold specificity for binding sites or allowed position shifts between different species (see the Materials and Methods section for a detailed description) are set by default to values that have been found to provide reasonable results. They can be modified by the user to optimize the results for other training sets.

In mode *Genmot*, the user should enter the training set CRM coordinates. The chosen evolutionary model for the TFBS should also be specified. The Felsenstein mode is computationally faster than the HB one. The results of the two modes have been found to be comparable (see Figures 2 and 3).

In mode *Scangen*, the algorithm scores and ranks intergenic sequences in the reference species, using a list of motifs, as described in the first ‘Results’ section and in ‘Materials and Methods’. The list of *de novo* *Genmot* motifs can be used as input. The user can set the length of the ranked sequences (1 kb is the default value) and the number of scoring motifs (5 is the default value). The default values have been chosen for computational efficiency but changes can improve results (see Supplementary Figure S3).

An example of *Imogene* output is displayed in Figure 6. The *Genmot* mode creates from the provided training set a list of ranked motifs together with their significance and over-representations (see the Materials and Methods section). The positions of these motifs on the CRM of the training set and on their homologous sequences in other species are also provided, as illustrated in Figure 6A for two motifs. Figure 6B shows the output of the *Scangen* mode for these two motifs. The ordered list of best-ranking intergenic sequences is given together with information on the closest TSSs.

## DISCUSSION

We have presented *Imogene*, a set of statistical tools and a computer software able to predict *de novo* relevant motifs in a moderate size set of functionally related CRMs and able to infer novel CRMs with a low FPR in both *Drosophilae* and mammalian genomes. In contrast to methods dedicated to the general discovery of CRMs (see 21^56^ for recent reviews and assessment), the task requires to extract specific information from a training set that by itself offers only weak statistically discriminative power. This challenge was previously tackled by ‘motif-blind’ methods (22) that aimed to characterize the statistical distribution of short sequences in the training without providing information on specific TFBS. *Imogene* makes use of a different strategy to work efficiently from a CRM set of modest size. It systematically exploits the information available in multiple sequenced genomes with a mode of motif inference that makes intrinsic use of quantitative models for binding site evolution. This leads it to achieve a performance for CRM identification comparable to ‘motif-blind’ algorithms (22) or even superior when motif conservation between different species is used as the CRM identification stage. But, in contrast to ‘motif-blind’ methods, *Imogene* also provides specific information on TFBS, an element of particular biological interest as well as a crucial ingredient for further biological tests of bioinformatic predictions.

*Imogene* relies on conservation between different species both as filtering step and to enlarge its training set. Phylogenetic conservation between multiple sequenced genomes has previously been shown to provide useful information on *cis*-regulatory motifs (57–59). Although many binding sites are not conserved (60), methods that use conservation among multiple genomes were found superior to single genome methods in a recent assessment of methods devoted to general CRM prediction (56). A simple peak phastCons score (61) was in fact found to be surprisingly efficient (56). In addition, ultraconservation has been found to reliably point out functional CRMs (62) in transgenic assays although subsequent deletion of these CRMs did not result in a marked phenotype (63) perhaps because of redundancy or too crude experimental assays.

Phylogenetic conservation, however, cannot *per se* address the question of specific spatio-temporal expression. The necessary information is provided to *Imogene* by the training set of CRMs with well-characterized expression. *Imogene* aim is to extract it optimally by making full use of several sequenced genomes, instead of focusing on a single genome (32) analysis, simply comparing the reference genome with another (64–66) or simply adding orthologous sequences (67). Similarly to the *Monkey* algorithm of (28) *Imogene* uses a model for the evolution of motif binding sites to properly weigh this additional information. The two algorithms are however complementary since *Imogene* creates *de novo* motifs from the training set while *Monkey* tests already well-characterized binding motifs.

The algorithm that lies at *Imogene* core was previously applied to gene co-regulation in *Drosophila* (14). Motifs predicted to be important for sensory-organ-precursor development were confirmed by site-directed mutagenesis. A significant fraction of top predicted new CRMs based on this predicted motifs were also shown to direct expression in SOP or more generally in the peripheral nervous system. The interest of predicting motifs *de novo* was further illustrated by a subsequent application of the algorithm to epidermal morphogenesis and trichome development in *Drosophila* (68). The algorithm provided a refined PWM for the master regulator Ovo/Shavenbaby and predicted as well a functionally important novel motif.

In spite of its successful application to gene co-regulation in *Drosophila*, it was not clear that the method could be successfully extended to decipher *cis*-regulatory information in the notoriously more difficult case of mammalian gene expression. We have provided here bioinformatics evidence that our developed algorithm indeed provides meaningful results in this case also. *Imogene* was shown to successfully recognize CRMs belonging to neural and limb development programs solely based on motifs that it has constructed *de novo* from the analysis of other CRMs. Furthermore, the created PWMs appear to comprise both known and new motifs, in strong analogy with the previous studied cases in the fly.

There are currently numerous cases for which a small number of CRMs belonging to the same program of gene expression has been characterized. At the same time, a large number of PWMs remain to be found. This is even more the case for CRMs. Therefore, the use of *Imogene* with its *de novo* motif building ability and allied CRM identification should provide helpful service to the community.

We have further shown that *Imogene* can discriminate between classes of CRMs, a capability that is clearly distinct from general CRM prediction (56). In this task, *Imogene* should usefully complement ChIP-seq data that are currently obtained for many developmental programs. Whereas ChIP-seq provides information on the binding of already known factors, *Imogene* is able to propose new motifs and helps to identify new involved DNA-binding cofactors and their binding sites. We anticipate that *Imogene* CRM discriminative ability is likely to be important for future studies of transcription regulation specificity in closely related cell types (e.g. different neuronal cell types) since even large-scale studies will probably not provide more than a few tens of differentially activated CRMs, the training set size targeted by *Imogene*.

We thus believe that *Imogene* is a useful addition to existing algorithms and softwares (32). We hope that it will serve as a helpful and timely tool in the difficult deciphering of gene regulation in higher eukaryotes.

## Supplementary Material

SUPPLEMENTARY DATA
