# Supplementary material for: Imogene: identification of motifs and cis-regulatory modules underlying gene co-regulation
Source: Nucleic Acids Res. 2014 Mar 25;42(10):6128–45. doi: 10.1093/nar/gku209 (PMC4041412; doi:10.1093/nar/gku209)
Supplement: SUPPLEMENTARY DATA [file supp_42_10_6128__index.html]

SUPPLEMENTARY DATA 

# Imogene: identification of motifs and *cis*-regulatory modules underlying gene co-regulation

## SUPPLEMENTARY DATA

**Files in this Data Supplement:**

- SUPPLEMENTARY DATA
