# Supplementary material for: Imogene: identification of motifs and cis-regulatory modules underlying gene co-regulation
Source: Nucleic Acids Res. 2014 Mar 25;42(10):6128–45. doi: 10.1093/nar/gku209 (PMC4041412; doi:10.1093/nar/gku209)
Supplement: SUPPLEMENTARY DATA [file supp_gku209_nar-00525-n-2014-File011.pdf]

Imogene: identification of motifs and cis-regulatory modules underlying gene co-regulation

Hervé Rouault, Marc Santolini , François Schweisguth, Vincent Hakim

February 25, 2014

Supplementary Figures

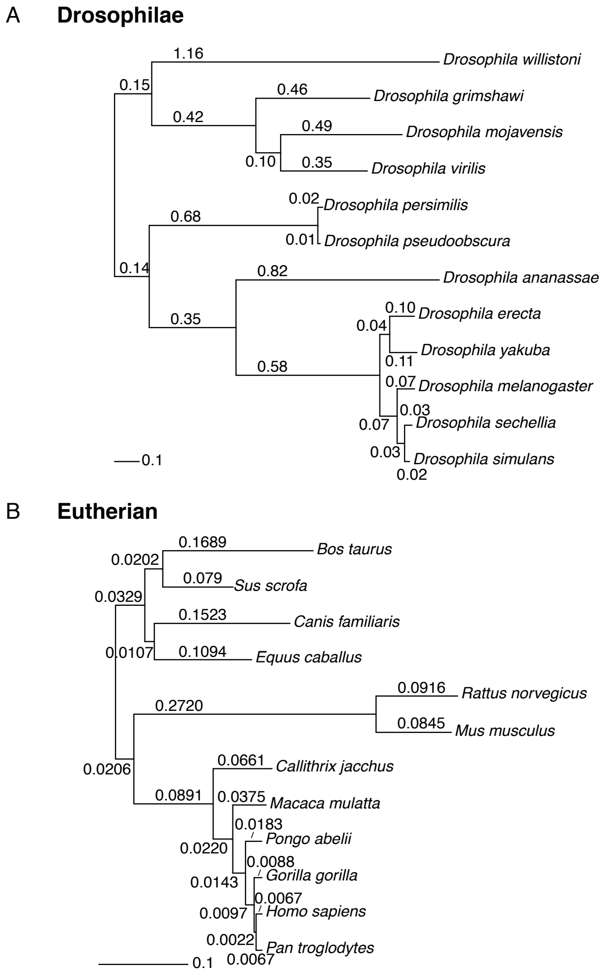

Figure S1. Phylogenetic trees and phylogenetic distances used by *Imogene*. The branch lengths represent the evolutionary distances d used by the evolutionary models at the motif construction stage.

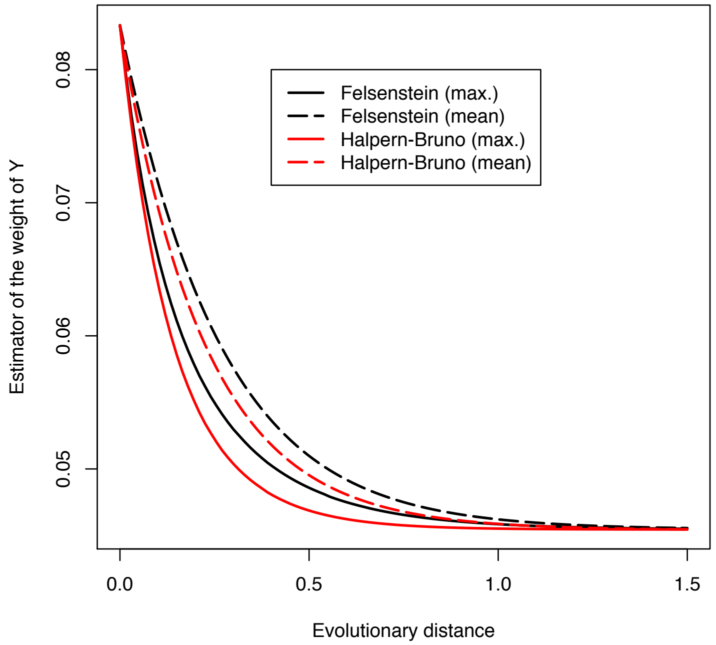

Figure S2. Simple example of motif inference with Felsenstein and Halpern-Bruno evolutionary models The inference of an ancestral base is compared in the simple case of two species at a phylogenic distance d from their common ancestor, for a two nucleotide alphabet,X and Y. The mean and maximum likelihood estimate of observing Y in the common ancestor given that the two species share an X is shown as a function of evolutionary distance d, for the Felsenstein or Halpern-Bruno evolutionary models. The likelihood is always smaller with the Halpern-Bruno model, reflecting the model greater evolutionary rate.

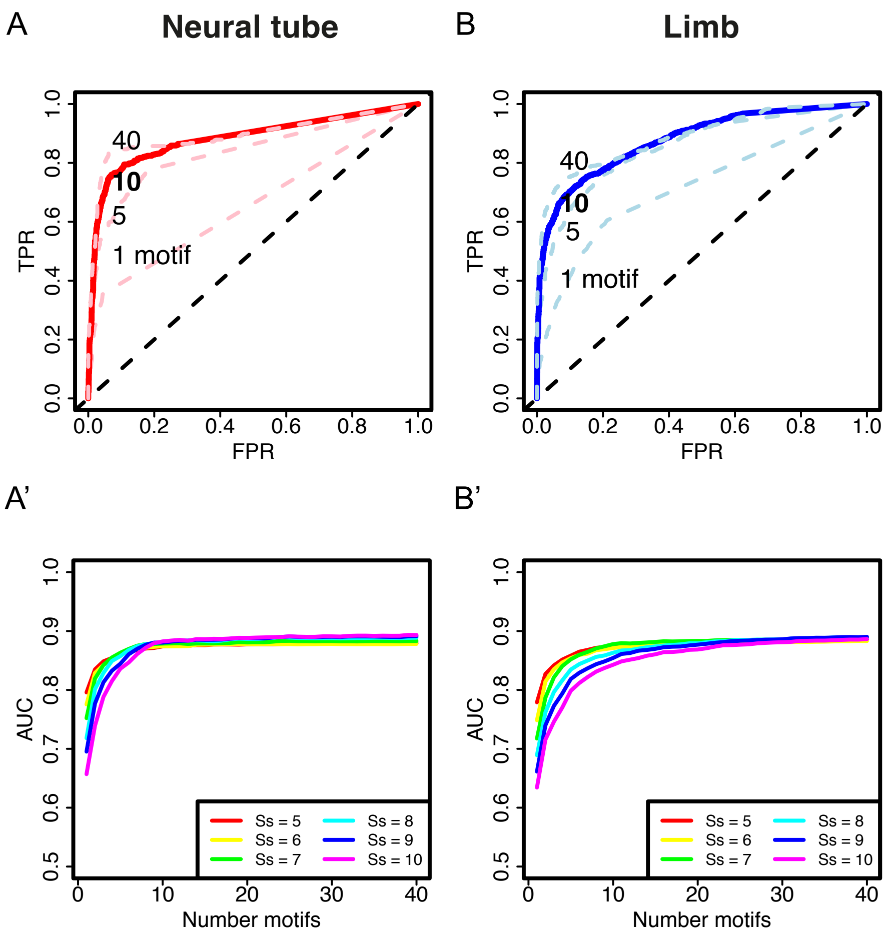

Figure S3. Dependence of the predictions on the number of scoring motifs ROC plots obtained at optimal scanning threshold using the Halpern-Bruno evolutionary model are shown for the neural tube (A) and limb (B) cases. Different curves are shown corresponding to sequences scored with different number of motifs: 1, 5 and 40 (light-color dashed lines), 10 (thick line). The ROC curves obtained for 10 motifs correspond to the ones shown in Fig. 3. To assess the degree of convergence, we computed the Area Under ROC Curve as a function of the number of motifs used (A',B',C'). We show the curves corresponding to the choice of different scanning thresholds S_s. In all cases, 10 motifs were sufficient for the AUC to reach convergence. The optimal S_s was chosen as the one maximizing the AUC for 10 motifs.

NEURAL		LIMB	
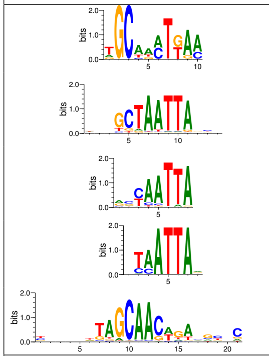	Motif 1	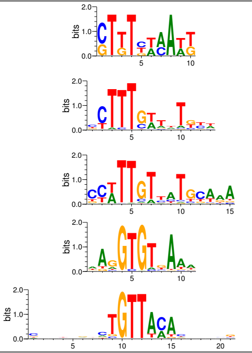	Motif 1
	CHX10_01 (Transfac)		TCF3_01 (Transfac)
	Nobox (Jaspar)		Sox2 (Jaspar)
	PDX1 (HT-SELEX)		TBX2 (HT-SELEX)
	Stp4 (UniPROBE)		Rdr1 (UniPROBE)
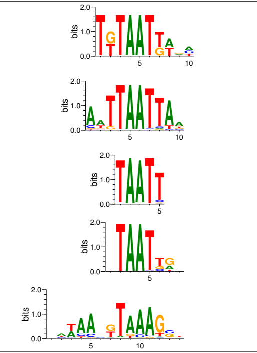	Motif 2	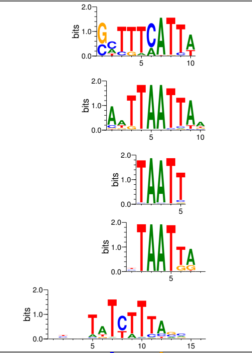	Motif 2
	LHX3_01 (Transfac)		LHX3_01 (Transfac)
	Prrx2 (Jaspar)		Prrx2 (Jaspar)
	BARX1 (HT-SELEX)		PDX1 (HT-SELEX)
	Irf3 (UniPROBE)		Egr1 (UniPROBE)
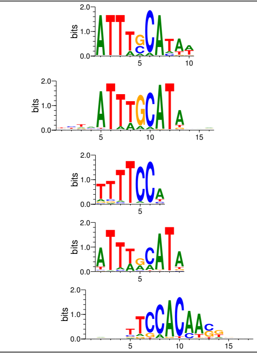	Motif 3	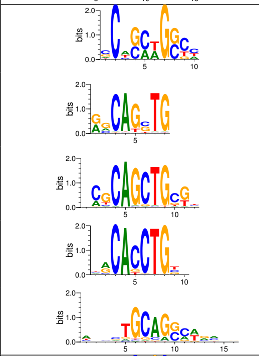	Motif 3
	OCT2_01 (Transfac)		MYOGENIN_Q6 (Transfac)
	NFATC2 (Jaspar)		NHLH1 (Jaspar)
	POU5F1P1 (HT-SELEX)		TCF4 (HT-SELEX)
	E2F2 (UniPROBE)		Osr2 (UniPROBE)
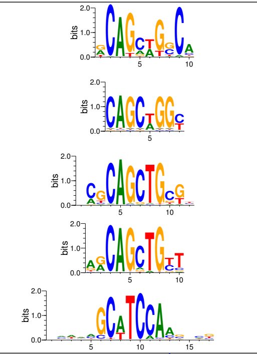	Motif 4	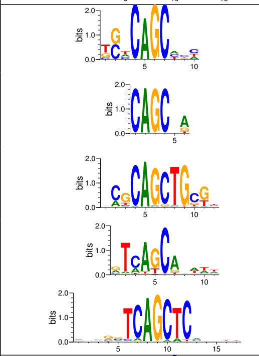	Motif 4
	HEB_Q6 (Transfac)		CBF1_QX (Transfac)
	NHLH1 (Jaspar)		NHLH1 (Jaspar)
	Ascl2 (HT-SELEX)		NRL (HT-SELEX)
	Hmbox1 (UniPROBE)		Zbtb3 (UniPROBE)
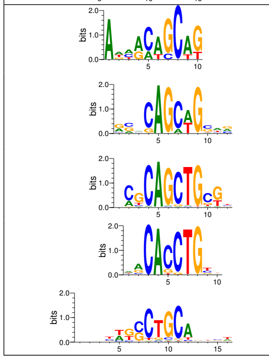	Motif 5	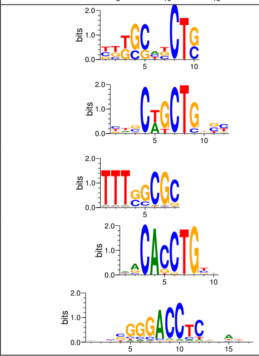	Motif 5
	NEUROD_02 (Transfac)		NEUROD_02 (Transfac)
	NHLH1 (Jaspar)		E2F1 (Jaspar)
	TCF3 (HT-SELEX)		TCF3 (HT-SELEX)
	Osr1 (UniPROBE)		Hbp1 (UniPROBE)

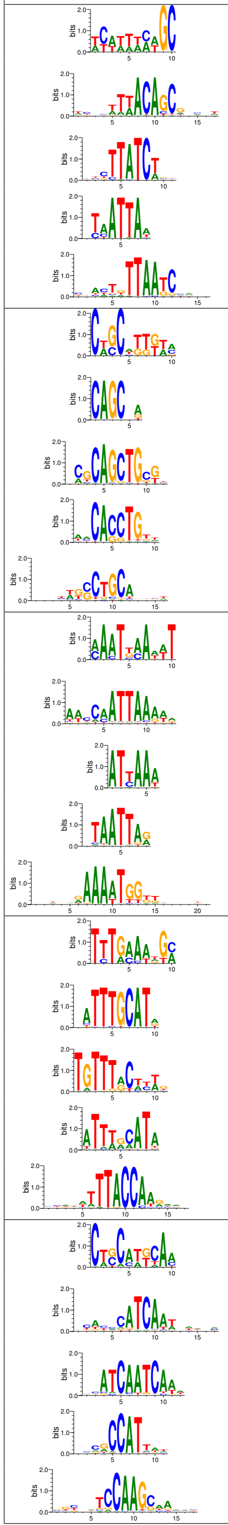

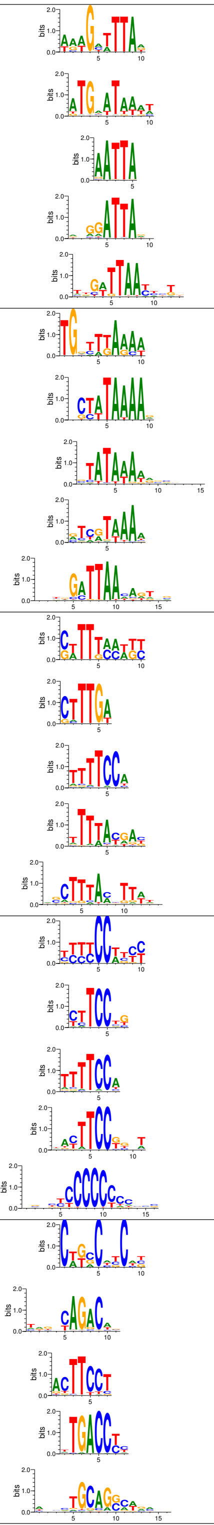

NEURAL	LIMB
Motif 6	Motif 6
RHOX11_01 (Transfac)	POU1F1_Q6 (Transfac)
Gata1 (Jaspar)	Prrx2 (Jaspar)
NKX6-1 (HT-SELEX)	GSC (HT-SELEX)
Sfpi1 (UniPROBE)	Elf3 (UniPROBE)
Motif 7	Motif 7
CBF1_QX (Transfac)	TATA_C (Transfac)
NHLH1 (Jaspar)	TBP (Jaspar)
FIGLA (HT-SELEX)	Hoxd9 (HT-SELEX)
Osr1 (UniPROBE)	Sfpi1 (UniPROBE)
Motif 8	Motif 8
NKX61_01 (Transfac)	LEF1_Q2 (Transfac)
ARID3A (Jaspar)	NFATC2 (Jaspar)
VSX2 (HT-SELEX)	Hoxd9 (HT-SELEX)
Rph1 (UniPROBE)	Irf3 (UniPROBE)
Motif 9	Motif 9
OCT1_B (Transfac)	ETS2_Q6 (Transfac)
FOXA1 (Jaspar)	NFATC2 (Jaspar)
POU5F1P1 (HT-SELEX)	Elf5 (HT-SELEX)
Pitx1 (UniPROBE)	Zfp740 (UniPROBE)
Motif 10	Motif 10
PBX1_04 (Transfac)	SMAD_Q6_01 (Transfac)
PBX1 (Jaspar)	SPI1 (Jaspar)
YY1 (HT-SELEX)	NR2F1 (HT-SELEX)
Tcf1 (UniPROBE)	Osr2 (UniPROBE)

Figure S4. Motifs learnt on the full training sets. The 10 best ranking motifs generated on the CRMs training sets are shown together with the closest motifs from the lists of TRANSFAC, JASPAR, HT-SELEX and UniPROBE databases (see *Distance between motifs* in *Methods* for details of motif distance computation).

	Mot1	Mot2	Mot3	Mot4	Mot5	Mot6	Mot7	Mot8	Mot9	Mot10
ZIC4-ZIC1_9_91261697_91263041	2	0	1	0	0	0	2	1	1	0
TCF4(intragenic)_18_69658816_69660452	0	0	0	1	1	2	0	0	0	0
CEI-IRX1_13_72435297_72436784	3	4	2	2	0	3	0	1	3	2
NBEA(intragenic)_3_55768657_55770664	0	1	1	2	1	0	0	0	3	1
AKT3(intragenic)_1_179080168_179081586	2	1	1	3	2	1	2	0	2	0
FOXG1B-PRKD1_12_51291542_51292872	4	2	1	0	2	0	0	3	1	0
DACH1(intragenic)_14_98553917_98556433	5	0	2	4	0	2	3	1	3	2
FAM44A-CPEB2_5_42914188_42915270	1	0	1	3	1	1	2	0	1	0
IRX4-IRX2_13_73170587_73173631	0	0	0	2	0	0	0	0	4	0
EBF1(intragenic)_11_44469978_44471372	2	3	1	1	0	3	4	1	0	0
ATG4C-FOXD3_4_99240573_99241457	0	0	0	0	0	0	0	0	0	0
CYLD-SALL1_8_91462919_91464123	0	0	1	1	1	0	0	0	1	0
POU2F1(intragenic)_1_167864366_167866439	5	0	4	1	0	0	0	3	0	3
APG4C-FOXD3_4_99040833_99042291	0	0	0	0	0	0	0	0	0	0
MGC14798-HH114_2_115363420_115365044	2	2	2	0	1	0	0	2	0	0
MGST1-LMO3_6_138199417_138201368	5	1	1	1	1	3	0	3	1	1
APG4C-FOXD3_4_98961102_98962673	2	2	2	2	3	1	4	0	0	0
FLJ46321-RASEF_4_73149468_73150526	0	0	2	4	0	1	1	1	0	1
TCF12(intragenic)_9_71823775_71824538	1	0	0	1	2	0	1	0	0	1
BMPER(intragenic)_9_23182371_23184296	2	1	1	1	0	2	1	0	1	0
SOX21-ABCC4_14_118834760_118836087	1	6	2	1	3	0	1	3	3	2
FANCL-BCL11A_11_25256346_25257683	0	2	1	0	3	0	2	0	0	0
DERA(intragenic)_6_137772070_137773298	1	5	0	1	1	1	2	0	0	0
MRPS9(intragenic)_1_42945168_42946091	1	1	0	2	1	1	1	0	0	0
YTHDF3-BHLHB5_3_16776170_16778776	2	2	0	1	0	0	0	0	1	0
STXBP6-NOVA1_12_47121350_47122759	1	4	2	3	0	2	2	0	0	0
IDH3B-CPXM1_2_130177541_130178125	0	0	0	0	0	0	0	0	0	0
LOC347487-SOX3_X_57972482_57973750	3	0	1	2	1	1	2	2	1	3

Figure S5. Neural CRMs and motifs. List of the neural CRMs used in this study. The number of motifs of different types on each CRM is given for the 10 best-ranking neural motifs shown in Figure S4.

	Mot1	Mot2	Mot3	Mot4	Mot5	Mot6	Mot7	Mot8	Mot9	Mot10
hs1435_7_106105018_106107143	1	1	2	2	0	3	3	0	3	0
hs126_14_97485454_97486724	5	1	2	0	0	2	1	3	1	0
hs1477_2_59400401_59401189	2	0	1	1	2	1	1	1	1	0
hs521_1_91610325_91611486	0	1	2	4	0	1	0	0	8	0
mm422_2_4477190_4478921	0	0	1	1	0	0	0	0	0	0
hs1432_13_91326599_91329775	0	0	0	1	0	0	0	0	0	0
hs1433_3_30003454_30008202	8	4	8	5	5	5	4	5	6	1
hs208_9_100171947_100173392	2	2	3	3	5	1	1	1	4	2
hs1507_1_75765578_75770167	1	0	5	4	3	0	1	0	7	1
hs774_3_5329674_5330756	4	2	1	0	0	2	0	2	0	0
hs919_15_50496379_50498196	3	1	1	0	2	1	1	1	2	3
hs326_19_45568075_45569359	1	0	4	1	2	3	3	0	0	1
hs72_8_91978407_91979282	1	1	2	3	2	2	0	0	2	1
hs1484_4_97888231_97891318	0	1	0	0	2	0	0	1	1	0
mm423_2_4508631_4509808	0	0	1	0	0	0	0	0	0	0
mm428_5_38308981_38309833	0	2	1	0	0	0	1	0	4	0
hs741_3_66874217_66875516	4	2	1	0	1	2	0	2	1	0
hs1148_12_119941220_119942766	0	0	1	0	0	0	0	0	0	0
hs1109_13_79503055_79504129	2	1	1	1	0	1	1	0	1	0
hs2041_9_96280544_96283360	2	0	0	0	0	0	1	0	0	0
hs1473_13_56260379_56262548	1	1	7	1	8	0	0	0	1	0
hs1434_14_23833434_23842485	1	1	7	5	3	0	4	2	4	3
hs1465_6_51144711_51148222	0	2	6	3	1	1	1	0	3	0
mm94_6_122342623_122346341	0	0	2	1	1	0	0	0	3	0
hs1452_10_45612931_45614502	0	0	0	0	0	0	2	0	1	2
hs1468_10_125358093_125366026	0	0	1	0	0	1	0	0	0	0
hs1586_13_15640807_15642666	0	1	1	1	1	2	0	0	3	0
hs1273_12_9344323_9346407	2	2	2	1	3	4	1	4	3	1
hs1278_2_137073444_137074711	1	1	5	3	0	0	0	1	0	1
hs1500_14_22281464_22282917	0	0	4	1	2	0	0	0	2	0
mm458_15_63025492_63026343	2	0	1	0	0	1	0	0	4	0
hs388_12_26576441_26577229	4	4	2	2	1	0	0	0	0	1
hs1491_14_25804749_25806653	1	0	6	0	6	0	0	0	3	3
hs1428_3_99469238_99471067	0	2	4	2	2	0	1	0	3	0
hs1430_6_52917020_52919645	5	1	4	1	2	1	1	0	2	0
hs1475_16_72685882_72688547	0	0	1	0	1	0	1	4	0	1
hs1448_2_171555881_171562133	1	3	5	1	0	0	1	0	1	0
hs644_12_34884495_34885741	0	5	4	1	0	1	1	0	2	0

Figure S6. Limb CRMs and motifs. List of the limb CRMs used in this study. The number of motifs of different types on each CRM is given for the 10 best-ranking limb motifs shown in Figure S4.

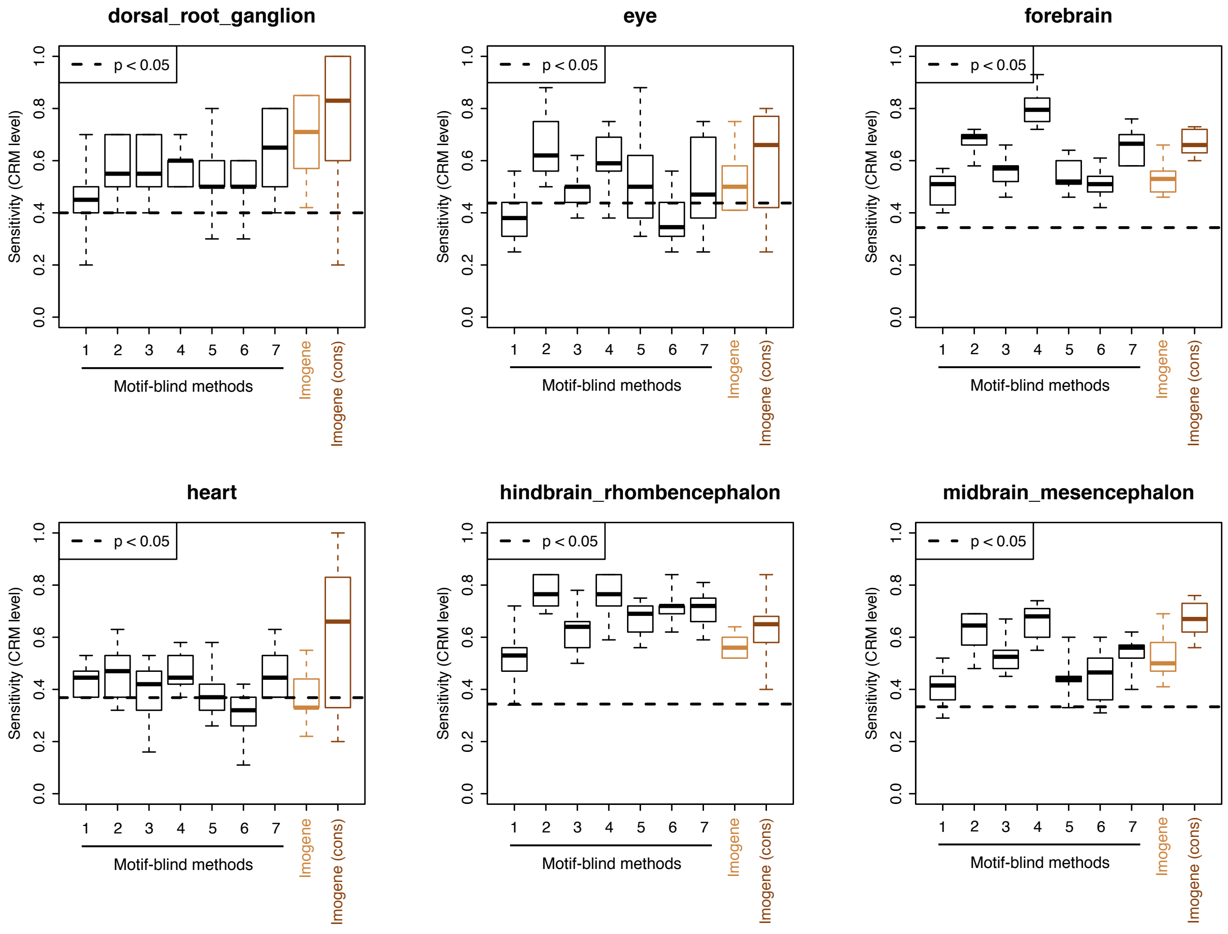

Figure S7. Comparison between *Imogene* and 'motif-blind' algorithms. Same as Figure 3C, D for the other test sets used in ref. [22] of the main text.

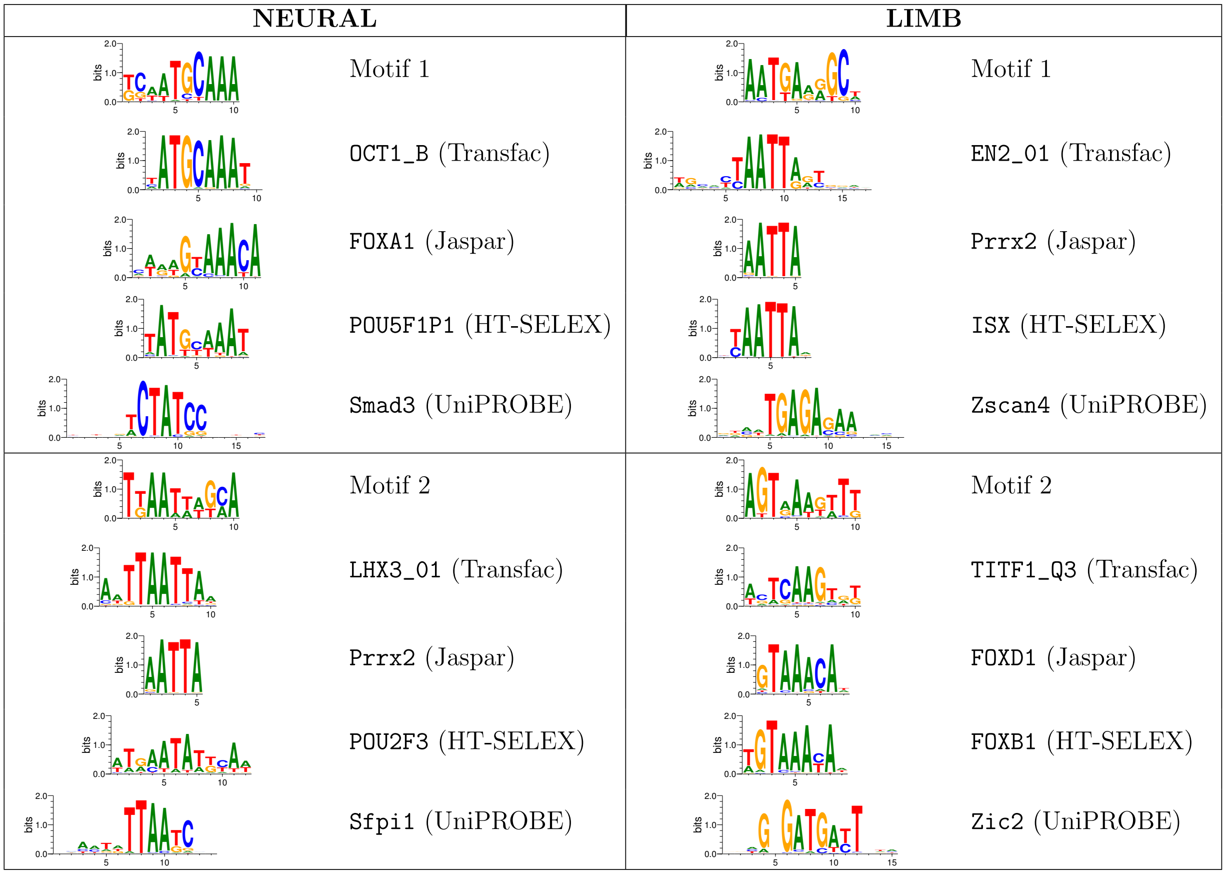

Figure S8. Motifs created by *Imogene*. for the discrimination of neural and limb CRMs. *Imogene* motifs shown in Figure 4 are displayed together with the closest motifs in the TRANSFAC, JASPAR, HT-Selex (ref. [50] of the main text) and UniPROBE (ref. [51] of the main text) lists of motifs.
